# Organic Sonochemistry: A Chemist’s Timely Perspective on Mechanisms and Reactivity

**DOI:** 10.1021/acs.joc.1c00805

**Published:** 2021-06-22

**Authors:** R. Fernando Martínez, Giancarlo Cravotto, Pedro Cintas

**Affiliations:** † Department of Organic and Inorganic Chemistry, Faculty of Sciences, and IACYS-Green Chemistry and Sustainable Development Unit, University of Extremadura, 06006 Badajoz, Spain; ‡ Dipartimento di Scienza e Tecnologia del Farmaco, Universita degli Studi di Torino, via P. Giuria 9, Torino 10125, Italy

## Abstract

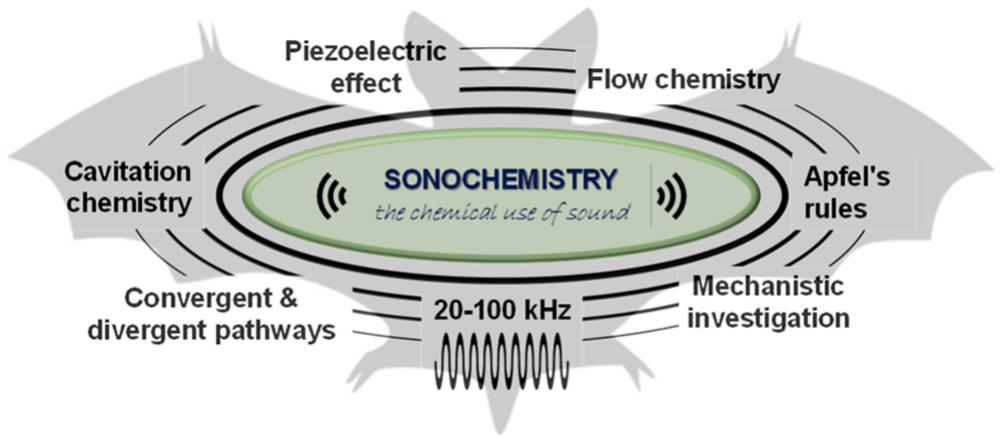

Sonochemistry, the use of sound waves, usually within the ultrasonic range (>20 kHz), to boost or alter chemical properties and reactivity constitutes a long-standing and sustainable technique that has, however, received less attention than other activation protocols despite affordable setups. Even if unnecessary to underline the impact of ultrasound-based strategies in a broad range of chemical and biological applications, there is considerable misunderstanding and pitfalls regarding the interpretation of cavitational effects and the actual role played by the acoustic field. In this Perspective, with an eye on mechanisms in particular, we discuss the potentiality of sonochemistry in synthetic organic chemistry through selected examples of past and recent developments. Such examples illustrate specific controlling effects and working rules. Looking back at the past while looking forward to advancing the field, some essentials of sonochemical activation will be distilled.

*“Alfred L. Loomis, Wall Street banker and scientist, with a beautifully equipped research laboratory at his Tuxedo Park residence where he cooperates in pure science necromancy with his friend, Professor R. W. Woods, of Johns Hopkins University, made public yesterday details of a new form of sorcery, super-audible sounds.”*

The New York American (1927)^1^

## Introduction and Motivation

Nearly one century after the seminal discovery by Loomis and associates of chemical, physical, and physiological effects induced by ultrasonic frequencies, sonochemistry has become a promising and cost-effective technique to reconsider sustainable chemical syntheses in line with the ever-increasing impact of environmental concerns. Sonochemistry has moved from a specialized field to inter- and multidisciplinary domains ranging from nanotechnology to drug delivery, among others. Although the use of ultrasonic activation has long been brought into the mainstream of organic synthesis, modern synthetic challenges such as selective functionalization, stereochemical control, or mechanistic interpretations still represent unsolved and/or open questions.

Compared to other enabling techniques, namely photochemistry, electrochemistry, and microwave chemistry, for which some variables are known and should be adjusted; sonochemistry is often viewed as a *black art*, where it is less easy to predict what you are going to get. Ways of thinking with this technique lie invariably in cavitation (*vide infra*), which is complex enough to model or interpret all effects in solution. This is reminiscent of the renaissance of sonochemistry in organic synthesis in the early 1980s as narrated by one pioneer, Jean-Louis Luche, who attempted a difficult Grignard reaction in a cleaning bath when all else failed. The transformation succeeded and led to new avenues and strategies in metal activation and improved one-pot Barbier-type reactions that bypassed tedious and hazardous organometallic reactions.^2^ Readers are referred to recent books and reviews that address the multiple applications of ultrasonics and sonochemistry and provide sufficient knowledge for further research.^3−7^ Previous textbook-like monographs, suitable for beginners and practitioners alike, deal with key concepts and useful tips while illustrating the broad applicability of ultrasonic waves.^8^ In fact, sonochemistry owes credit to some pioneers who paved the way to the current state of the art, both demystifying the technique and showing the fundamentals embedded in acoustics along with working rules. To name a few who influenced our approach to organic sonochemistry, but by no means excluding others: Jean-Louis Luche, Ken Suslick, Tim Mason, Arnim Henglein, Peter Riesz, Takashi Ando, Oleg Abramov, and Jacques Reisse.

Herein, we concentrate on sono-organic methods and general trends, which reflect well-established points and open up prospects for future explorations, especially in organic and medicinal chemistry. We begin with some introductory aspects of cavitational effects, followed by practical remarks on common ultrasonic reactors and how a series of external parameters, often overlooked or misunderstood, matter. The Perspective continues with some empirical rules, which show the opportunities and challenges for achieving creativity and selectivity under eco-friendly conditions. We finally address future orientations and innovations, such as piezo-redox catalysts, flow designs, and in-line automation.

## What Is Cavitational Chemistry?

Ultrasound refers to inaudible acoustic waves, which are generally considered as being about 20 kHz, even though the audible upper limit of frequency in humans depend on age and other physiological conditions. Power ultrasound is devoted to high-intensity applications that produce permanent changes in the physical and chemical properties of materials. Most sonochemical reactions are conducted at ultrasonic frequencies between 20 and 100 kHz with intensities high enough to cause cavitation in the liquid medium. This unique phenomenon provides the kinetic energy that fuels a transformation to completion and involves a juxtaposition of purely chemical (i.e., bond-breaking and bond-forming reactions) and mechanical (cleaning, dispersion, friction, interface instability, degassing, defoaming, and others) effects. Within this context, ultrasound-induced chemistry can be appropriately defined as *cavitational chemistry*.^9,10^ Cavitation refers to the generation of voids or microsized cavities when a liquid suffers a sufficient pressure drop that disrupts its cohesive forces. The subsequent and violent collapse to restore the intermolecular interactions releases a huge energy input accounting for the aforementioned effects. Although cavitation can be produced by various methods, there is little doubt that acoustic cavitation is the best known. The extreme conditions inside the microbubble with temperatures of several thousand Kelvin and more than 1000 atm were notably advanced by Lord Raleigh in the first hydrodynamic model as early as 1917.^11^ Given the small size (∼1 μm) and transient lifetime (∼1 ns) of bubbles, the physical nature of cavitation has been ascertained through light emission spectra (sonoluminescence) that occur during bubble collapse, which have even unveiled formation of plasma-like conditions in recent studies.^12−14^

Surprisingly, the first chemical effects observed by Loomis and his co-workers were ascribed vaguely to “frequency effects” (they employed high-frequency transducers, unusual by that time). By 1929, however, the first description of ultrasonic oxidations suggested the putative role of hydroxyl radicals.^15^ Richards reported the first comprehensive review of supersonic transformations, including sonochemical ones, in 1939 with 348 references.^16^

**Figure 1:**
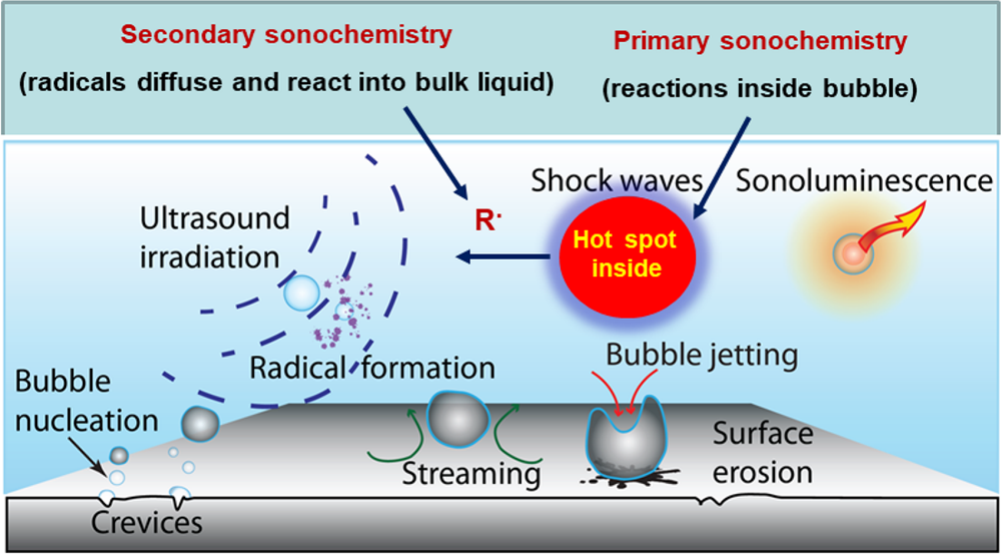
Representation of chemical and mechanical effects induced by acoustic cavitation.

A catching picture of cavitation and its effects is shown in Figure 1. Volatile reagents and solvents will be trapped in the microbubbles, then undergoing homolytic cleavage and/or conversion into excited states. This represents a primary sonochemistry that can be followed by secondary effects, as such reactive species delivered into the liquid will react with molecules at the vicinity of the collapsing bubble or recombine to form stable products. Bubble collapse is accompanied by shock waves and shear forces that may cause further rupture of nonvolatile molecules accumulated at the bubble interface. In heterogeneous reactions involving solid–liquid and liquid–liquid interfaces, such forces result in high-velocity flows, acoustic streaming, which enhance both mass and energy transfers. Bubble implosion on surfaces generates microjets, hammering, and interparticle collisions,^17^ all accounting for the well-established mechanical action and cleaning of acoustic cavitation.^8,18^ In aqueous media and in the presence of oxygen, formation of reactive oxygen species (ROS) will take place, with the OH radical being the most significant and nonspecific oxidizing agent. Accordingly, a large variety of organic molecules, especially pollutants, can be degraded by sonication alone or combined with other protocols.^19^ Good correlations between octanol–water partition coefficients and reaction rates have been observed, as hydrophobic pollutants tend to accumulate near cavitation bubbles.^20^ The cytotoxicity associated with some ROS can also be harnessed in sonodynamic therapies, involving the action of sonosensitizers under ultrasonic stimulation.^21^

**Scheme 1:**
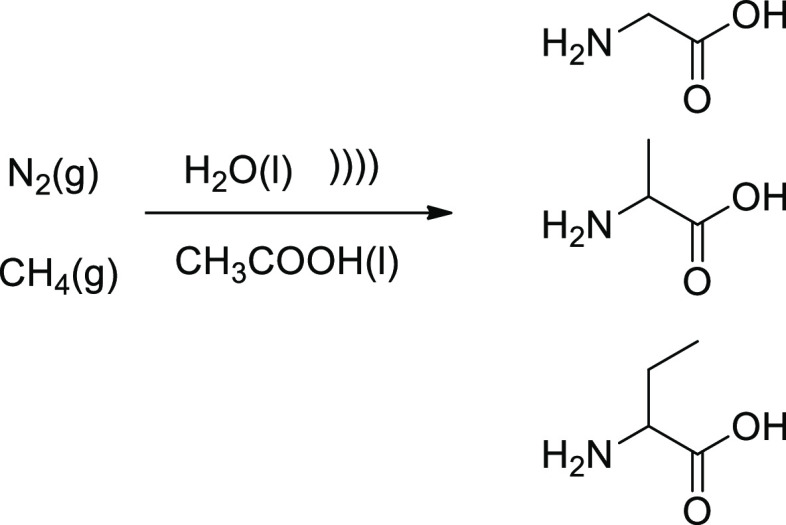
Prebiotic-like Cavitational Synthesis of Amino Acids

Remarkably, cavitation is a *geochemical phenomenon* that occurs naturally under intense hydrodynamic conditions, turbulent streams, earthquakes, or waterfalls and wherever a shear force rapidly disrupts the continuity of aqueous surfaces. Collapsing waves in primeval oceans would have generated sufficient cavitation to induce chemical reactions and hence, formation of organic compounds.^22^ The putative role of cavitation in prebiotic synthesis of life-based building blocks was suggested long time ago,^23,24^ but its potentiality has scarcely been explored. A recent and detailed study from Grieser’s team reported that cavitation may be effective in producing amino acid mixtures from carbon and nitrogen sources available in the early biosphere.^25^ Ultrasonic irradiation (355 kHz, 70 W) of a mixture of nitrogen, methane, water, and acetic acid led to formation of a few amino acids (glycine, ethylglycine, and alanine) at rates in the range 1–100 nM/min (Scheme 1).

It is believed that pyrolytic decomposition of substrates would occur within the imploding bubbles, affording H^•^, HO^•^, ^•^NH^•^, and NH_2_^•^ radicals, among others, with acetic acid being sufficiently volatile to enter an expanding bubble and fragmented into carbonaceous radicals as well. Both radical–radical and radical–molecule reactions should be equally probable given the high radical concentration within the bubbles and yielding the end products in solution. This sort of cavitational syntheses provides new vistas in origin-of-life studies, as evidenced recently by Zeiri et al. from a computational simulation of cavitational impacts (via shock wave modeling) on variable gaseous mixtures (CO, CO_2_, CH_4_ + N_2_, NH_3_, or HCN) showing the generation of prebiotic monomers like glycolaldehyde, glyceraldehyde, urea, cyanamide, formamide, isocyanic acids, and others.^26^

## Optimization and Modes of Operation. Apfel’s Rules

**Figure 2:**
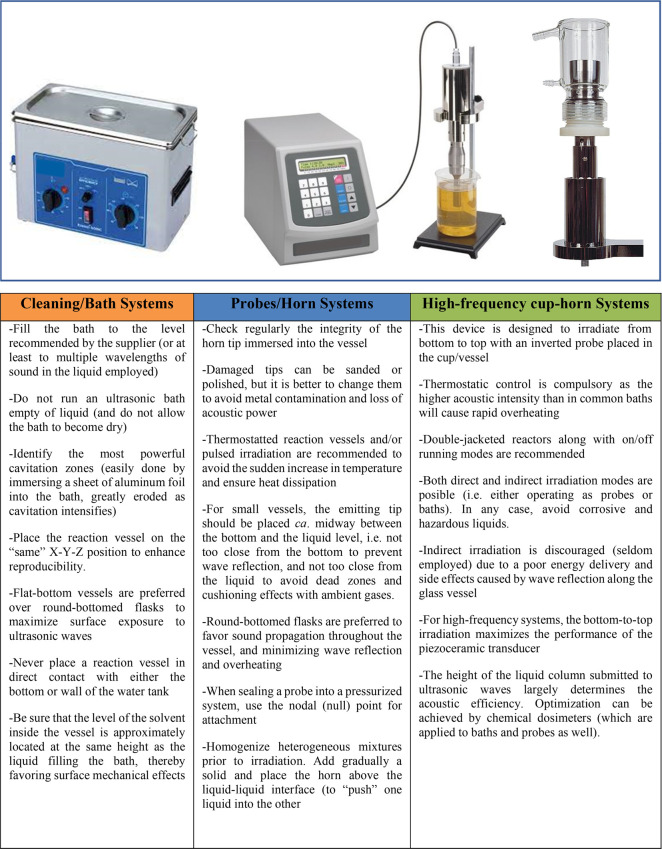
Some basic precepts and tips for proper use of ultrasonic devices. The image (from left to right) shows the usual setups (bath, probe, and cup horn) to conduct sonochemical reactions.

Although this Perspective is not intended to be a tutorial on ultrasonics, like in the case of raising techniques, electrochemistry, for instance,^27^ some background and guidelines are pertinent showing the simplicity of the technique through three common devices. The toolbox relies on the proper use of operational modes to ensure reproducibility, as gathered in Figure 2.^6,28^ Such setups usually work at a given frequency with wave amplitudes controlled by external voltage. Multifrequency reactors, even reaching the MHz zone, are more expensive and require a bit of expertise. Dosimetry methods to determine the actual acoustic power are strongly encouraged,^8^ especially with ultrasonic baths. Numerous practitioners report essentially the nominal frequency (generally between 25 and 40 kHz) and output power indicated by the supplier, which are largely meaningless. Nor do reviewers, unfamiliarized with sonochemistry, recognize the importance of determining a few working parameters. Since sound radiation is devoid of quantum character, similar chemical effects can be attained within a broad range of frequencies. Certainly, there are frequency effects, but they should be assessed separately as a function of the acoustic intensity. The solvent is more than the reaction medium. While conventional chemistry pays attention to solvent’s properties like acidity or polarity, physical characteristics (volatility, viscosity, or surface tension) play key roles in sonochemical activation as they will affect both sound propagation and cavitational implosion.

The design of devices enables various adjustments like pulsed on/off irradiation, pressurized vessels, or bubbling gas. Even if the hot-spot temperature inside the microbubbles will determine the outcome of a sonochemical reaction (amount and nature of excited species), the macroscopic temperature has the same relevance as in silent reactions. Pulsed mode or thermostated vessels provide accurate thermal control. A “quick and dirty” solution, yet practical with cleaning baths, involves the deliberate addition of ice or cold water as the temperature rises after prolonged sonication.

In the late 1980s and early 1990s, some sonophysicists, long engaged in cavitation, became rather perplexed when chemists reported sonochemical experiments, often using baths, without knowing the acoustic field to some detail.^29^ As mentioned above, this criticism is also pertinent in 21st century sonochemistry, although a full physical modeling of that field is not required and it is obviously beyond the scope of synthetic purposes. In context, however, one should recall a few precepts, postulated by Apfel some decades ago,^30^ which are known as *Apfel’s golden rules* and expressed in a Socratic style: “know thy liquid”, “know thy sound field”, and “know when something happens”. Apfel reasoned that the first determines the cavitational threshold, the second the accuracy of measurements, and the third tells us that the effects of cavitation indirectly inform about the magnitude of cavitation itself. In short, the rules warn about the measurement and significance of the acoustical parameters and, as a consequence, on the right use and calibration of ultrasonic devices.

The definition and use of physical parameters in the field of ultrasonics are rarely obvious to the chemist and can be the source of misinterpretations and overestimations. They are, however, of paramount importance for reproducibility and should remain constant for comparative analyses. Ultrasonic frequencies are divided into two broad groups: low-frequency/high-power (from *ca*. 18 kHz to 100 kHz) and high-frequency/low-power (from 100 kHz to 10 MHz). The former is the usual area of sonochemistry where mechanical effects are prevalent, while enhanced radical production and therefore chemical effects, are associated with the high-frequency range.^28,31^ The reactive species generated within the collapsing bubble and diffusing into the surrounding liquid, follow different evolution as a function of the bubble lifetime and radius, which depend among others on the frequency. Although global effects vary with the acoustic frequency,^32^ it makes no sense *to worry about using specific frequencies*. Moreover, in some common devices, such as baths, the resonance frequency is modified by other parameters like temperature, the level of liquid in the tank, and the transducer load owing to the reaction mixture.^8,28^

Since sound is caused by pressure waves, the acoustic intensity can be defined as the power per surface unit and denoted as
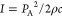
1where *P*_A_ is the acoustic pressure amplitude of the wave, ρ the density of the liquid, and *c* the speed of sound in that medium. This expression is valid only for planar or spherical waves generated within low-pressure variations. Cavitation at low-frequency ultrasound induces significant changes in acoustic pressures and complex, nonlineal behaviors are observed.^33^ A relative measurement (and usually invoked in chemical studies) can be obtained from the amplitude of the emitter. However, a common mistake is to *associate the ultrasonic power effect with the vibrational amplitude* because the acoustic power transmitted depends on the emitting surface. Even worse, if the amplitude is maintained at the same level during a chemical reaction, any modification of the system (such as temperature, viscosity, concentration, etc.) will also modify the power. In other words, the amplitude must change if the power is to remain constant. That said, running a reaction at different vibration amplitudes enables comparisons of synthetic efficiency, provided that other parameters, including the shape and dimensions of the probe assembly, are specified.

Probably the best way to characterize the energy delivered into the reactor is the notion of ultrasonic power, i.e., the transmitted acoustic power (in watts or milliwatts) or the power density (W/cm^2^). This value can be inferred from physical or chemical measurements.^8,28,34^ The easiest physical method implies the assimilation of the reaction vessel to a calorimeter, where the temperature is measured and plotted against time. Above the cavitation threshold, the acoustic energy is partially dissipated into heat. The calorimetric power (in watts) can be deduced from the following relationship with slope Δ*T*/Δ*t*
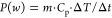
2where *m* is the mass of liquid irradiated with ultrasound and *C*_p_ the isobaric thermal capacity. It is obvious, by virtue of the nonlinear behavior of cavitation bubbles, that the bulk temperature may not be spatially homogeneous and variations occur within the reaction vessel.^35^ Accordingly, irradiation focused on small volumes is desirable in the search for reproducibility. Chemical dosimetry involves the estimation of radical species by sonolysis, which can be quantified by UV–vis or fluorescence measurements.^36^ In aqueous media, OH radicals are created and monitored by different methods, the most popular being the Weissler reaction, based on the oxidation of an aqueous KI solution to I_2_; addition of some halogenated solvents greatly enhances the oxidation rate.^8^ In organic solvents, radicals other than OH^•^ can be generated, and the decomposition of reagents like DPPH (2,2-diphenyl-1-picryl-hydrazyl) has become a very sensitive probe of organic sonolysis. Spin-trapping of the radicals with monitoring by ESR is an accurate method, not always available in every laboratory.^37^ More sophisticated and accurate protocols take advantage of sonoluminescence spectra or chemiluminescence mapping obtained by oxidative degradation of luminol.^38,39^ These methods provide a topology of the ultrasonic field, i.e., the spatial distribution of acoustic energy and location of maxima in cavitation.

## Sonochemical Reactivity: Convergent and Divergent Pathways

When we were introduced to the field of ultrasonics, our motivation as organic chemists was to understand an apparently new model of chemical reactivity, other than photochemistry and electrochemistry. Luche proved to be an efficient mentor and wondered about the central point:^2,10^*When and where does sonochemistry take place?*

**Scheme 2:**
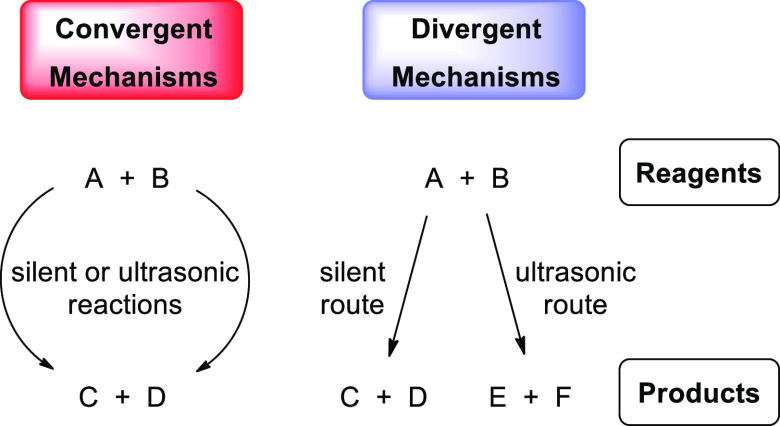
General Classification of Sonochemical Reactions Relative to Silent Conditions Depending on Product Distribution

A general misconception dealing with ultrasonic activation is the often unproven assumption that a radical pathway is triggered and single-electron transfer (SET) occurs, as cavitational implosion causes solvent’s pyrolysis and homolytic ruptures. The reaction partners can be unaffected and the whole reaction remains insensitive to sonication or exhibits a weak effect. This consideration is behind the so-called Luche’s rules in an attempt to determine whether or not the rate-limiting step is actually the sonication-sensitive step and, whether cavitation or new chemical intermediates dictate the reaction outcome. Luche himself cautioned about the general use of a classification based largely on empirical observations. There is a consensus on the *Second Class* reactions involving *heterogeneous ionic reactions*, both solid–liquid and liquid–liquid, where mechanical effects derived from violent bubble collapse at the interfaces (*vide supra*) account for rate and yield enhancements. Moreover, polar and nonvolatile molecules can accumulate at the bubble-liquid interface, yet experiencing breakage by shock waves. As we shall see shortly, the categorization is unclear in numerous cases and borderline situation emerge. When seeking a mechanistic rationale, a convenient approach is the elucidation of *convergent* and *divergent* processes (Scheme 2).^10,40^ This view on reactivity envisages the plausibility of competitive, simultaneous polar and radical pathways. When silent and ultrasonic reactions converge to the same product(s), only an overall acceleration usually results from sonication along with a different product distribution. If the two reaction pathways are divergent and afford different products, sonication carries a true chemical component that leads to switching. It is worth pointing out that *both convergence and divergence under sonication do not necessarily imply a competitive radical route*. This reminds us that the first sonochemical switching reported involved the change from an electrophilic aromatic substitution under stirring to a bimolecular nucleophilic substitution by sonication, as the more vigorous ultrasonic agitation alters the surface sites of the catalyst, thereby hampering the former reaction. This interpretation, now widely accepted, might not exclude the generation of radical species on a solid surface, provided the organic precursor is prone to undergo a SET pathway.

**Scheme 3:**
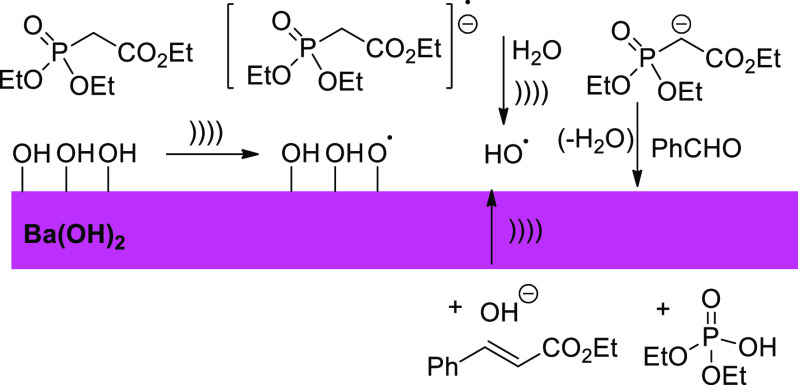
Convergent Sonochemical Wittig–Horner Reaction Catalyzed by Barium Hydroxide

A paradigmatic illustration of sonochemical convergence is a Wittig–Horner olefination catalyzed by Ba(OH)_2_, for which there is compelling evidence of a radical pathway with sonication playing a 2-fold role.^41^ The hydroxy groups on the catalyst surface serve as single-electron releasing sites which under ultrasound produce the radical anion of the phosphonate ester. The other ultrasonic effect is to cleave the trace amounts of water affording OH^•^ radicals in sufficient concentration to sustain the catalytic cycle (Scheme 3).

Homogeneous reactions in solution, illustrated by numerous addition and substitution reactions, appear to be the most problematic cases to understand. The hydrolysis of saccharose is a valuable example where the increase of reaction rate observed with irradiation is related to the cavitational event, as some formic acid, most likely generated by oxidation of the sugar with OH radicals, catalyzes this pH-dependent reaction. The conjecture could be confirmed by performing a mechanically stirred reaction in the presence of HCOOH with quasi-identical results as for the sonolyzed reaction.^42^ Acid-catalyzed hydrolysis of esters or acetalization reactions exhibit, however, weak sonication effects with only marginal increases in the rate. The fact that irradiation at higher frequencies does not modify the rate with respect to the nonsonicated reactions points to the negligible effect of radical production on such purely ionic mechanisms.^10^ Conversely, heterogeneous acetalization of unprotected sugars, which remain insoluble in the reaction mixture, are significantly accelerated by the sonication treatment.^43^ Clearly, the polar mechanism is not affected, but the enhanced mass transfer relative to conventional stirring leads to a greater yield. Notably, products are usually cleaner as acceleration avoids side reactions occurring in solution, and due to acid catalysis in a simultaneous way as the heterogeneous transformation proceeds. Such side reactions, being homogeneous polar processes, are not affected by sonication either.

**Scheme 4:**
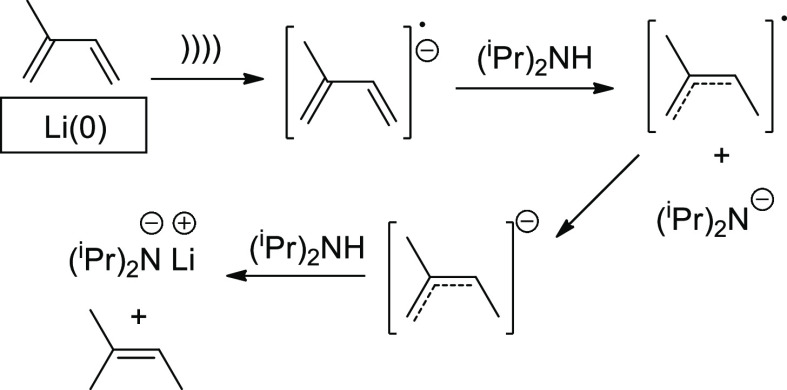
Preparation of LDA by Activation of Lithium with Ultrasound and Isoprene as Electron-Transfer Reagent

Substrates with ambident reactivity or those leading to stabilized carbon- or heteroatom-centered radicals represent the realm of the third class of sonochemical reactions, which can follow either SET reactions or conventional ionic/concerted routes. The direct coupling of metals and organic partners usually requires harsh conditions or are virtually negligible at room temperature, most likely owing to the heterogeneous nature of the system. Sonication in the presence of electron carriers (like benzophenone or 4,4′-di*tert*-butylphenyl affording almost instantaneous production of their colored radical anions) leads to dramatic reaction rates in the so-called *supersonic* preparation of versatile metal reagents.^44^ This result would not be expected on a mechanical effect alone and lends instead support to a SET pathway triggered by ultrasound. Two noticeable examples are lithium diisopropylamide (LDA) and samarium(II) iodide, widely employed in contemporary organic synthesis.^45,46^Scheme 4 depicts the expeditious sonochemical preparation of LDA that can be conducted in less than 30 min at room temperature and bypass the inert and dry conditions used conventionally.^47^ Sonication of diisopropylamine, lithium powder, and an electron carrier (ideally isoprene as the only byproduct is volatile methylbutene), in THF or a THF–heptane mixture, affords the desired LDA reagent. Electron transfer from the metal surface to the dialkylamine occurs almost immediately under sonication.

**Scheme 5:**
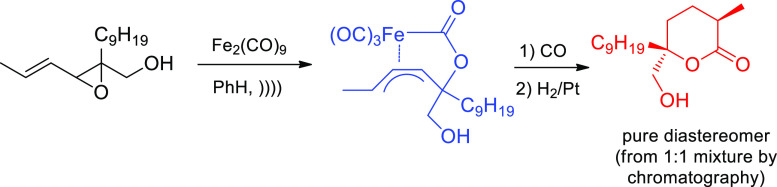
Short Synthesis of Malyngolide Involving the Sonochemical Formation of a Stable Ferrilactone

The ease with which some metal reagents are obtained by sonication can further be coupled with elegant elaborations of natural product skeleta, like the families of avermectins and milbemycins, especially aided by transition-metal carbonyls. Thus, π-allyl tricarbonyliron lactones (ferrilactones), which can be isolated as stable crystalline solids from a variety of carbonyl, epoxide, or aryl sulfone reagents, lead to four-, five-, and six-membered lactones and lactams. Ultrasound enables their transformation into more sensitive substances such as glycosides, glycals, and medium-ring cyclic ethers that are unavoidable under thermal conditions.^44,48^ As portrayed in Scheme 5, malyngolide (isolated from a 1:1 diastereomeric mixture by chromatography), a natural product present in blue algae and active against *mycobacteria* and *micrococcus*, could be easily prepared from a vinyl epoxide through a sonochemical reaction with Fe_2_(CO)_9_ in benzene. Sonication releases Fe(CO)_5_ or Fe(CO)_4_ that can be trapped by alkenes and other double bonds. Such coordinatively unsaturated metal species play the equivalent role of radicals or carbenes in all-carbon chemistry, and account for the positive effect in activating volatile metal carbonyls.

**Scheme 6:**
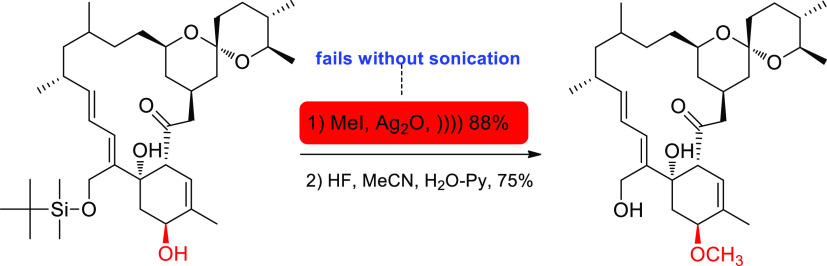
Semisynthetic Route to Enantiopure Milbemycin-β1 The first step fails without sonication.

**Scheme 7:**
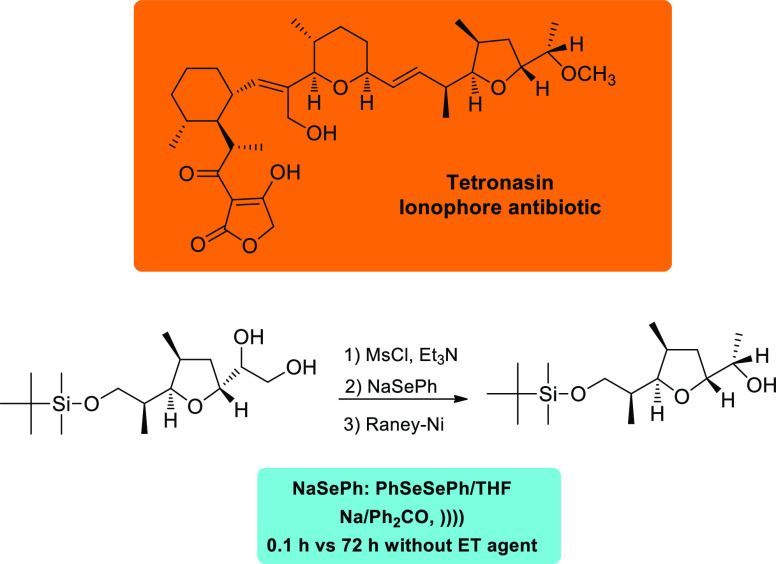
Key Intermediate’s Synthesis toward Tetronasin

It is somewhat counterintuitive that violent cavitation leading to turbulent hydrodynamic regimes achieves selective transformations, which would not occur under other conventional conditions. In the synthesis of (+)-milbemycin-β1, structurally related to avermectins and isolated from *Streptomyces* bacteria, and employed for veterinary use, a key alkylation step was only successful with ultrasound and compatible with a sensitive array of functional groups (Scheme 6). Likewise, the synthesis of naturally occurring bioactive compounds benefitted from the pluses of sonication, as exemplified in the synthesis of a key intermediate en route to tetronasin, an ionophore antibiotic (Scheme 7).^44^ The use of NaSePh in the second step, sonochemically generated by cleavage of PheSeSePh with sodium in THF, proved to be instrumental. In the presence of benzophenone as ET reagent, sonication speeds up the reaction from 72 h (without the ketone) to 0.1 h.

**Scheme 8:**
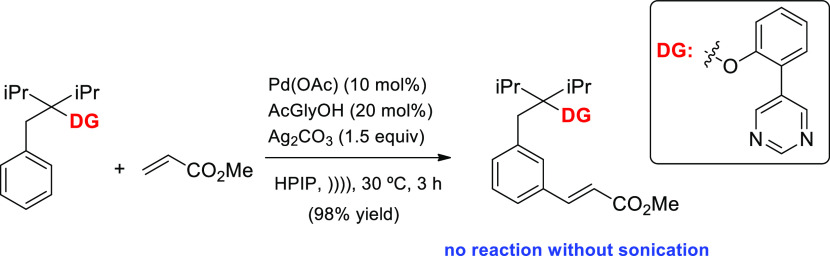
Direct and Selective Meta-C–H Substitution of Arenes Mediated by Ultrasound

These transformations take place under heterogeneous conditions and can reasonably be categorized within the Type-III class of sonochemical reactions capable of following either polar or radical mechanisms. Selectivity could then be ascribed to reactions occurring prevalently at the solid–liquid interface, rather than in solution, with adsorption–desorption promoted by cavitation. The argument is reinforced by other cases where both regio- and stereoselectivity are enhanced using low acoustic powers. Conversely, poor selection takes place as the ultrasonic intensity increases, thereby removing faster adsorbed species or transient intermediates that would evolve in the bulk liquid.^49^ The point is further witnessed by a recent and salient Pd-catalyzed *meta*-selective C–H functionalization of arenes tethered with a controlling distal group.^50^ Using a cleaning bath at room temperature without special power control, alkylation, olefination, acetylation or cyanation proceeded selectively at *meta*-position and without a trace of disubstituted *meta*-isomers observed under thermal reactions at higher temperature and longer reaction times (Scheme 8). Some reactions did not occur in the absence of sonication. The rationale should be involving a conventional metal-catalyzed arene substitution, although the assistance of acoustical force cuts the energy barrier and enhances a direct meta-functionalization.

**Scheme 9:**
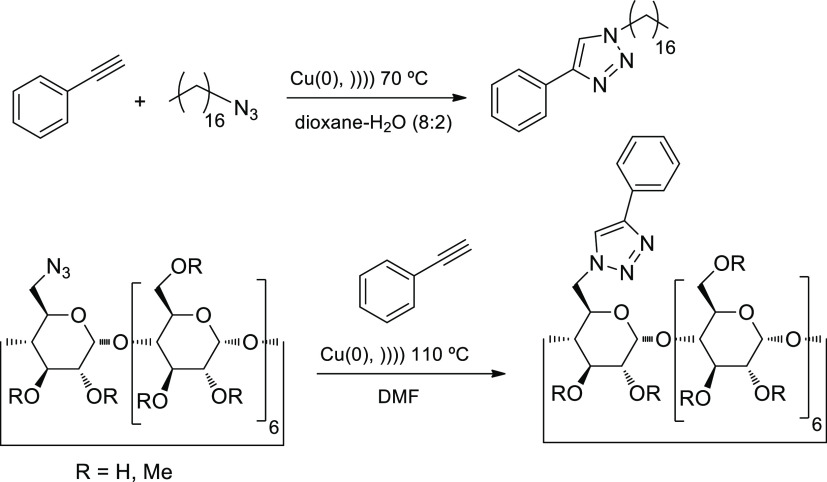
Alkyne–Azide 1,3-Dipolar Cycloadditions Activated by Sonication Using Metallic Copper

In retrospect, the main application of ultrasonically activated zerovalent metals is linked to one-step Barbier-type reactions, usually affording convergent results relative to silent reactions and employing more sustainable conditions such as aqueous media.^51^ SET mechanisms are plausible for electropositive metals and those having low ionization potential energy as surfaces become rapidly depassivated from hard and unreactive coatings. Cheap and less hazardous metals, rather than ionic compounds, have also been harnessed for orthogonal protocols, like the azide–alkyne cycloaddition. Thus, we were able to develop an improved click reaction using metallic copper that overcomes other limitations such as the in situ generation of Cu(I) species and the removal of copper salt byproducts.^52^ The Cu(0)-based protocol can be applied to simple and elaborated substrates (like cyclodextrins) and gives rise to products in higher yields and shorter times than the nonirradiated reactions. This also avoids the use of additional ligands to stabilize Cu(I). Polar solvents are required, which allow for higher cavitational energy coupled with heating (Scheme 9).

**Scheme 10:**
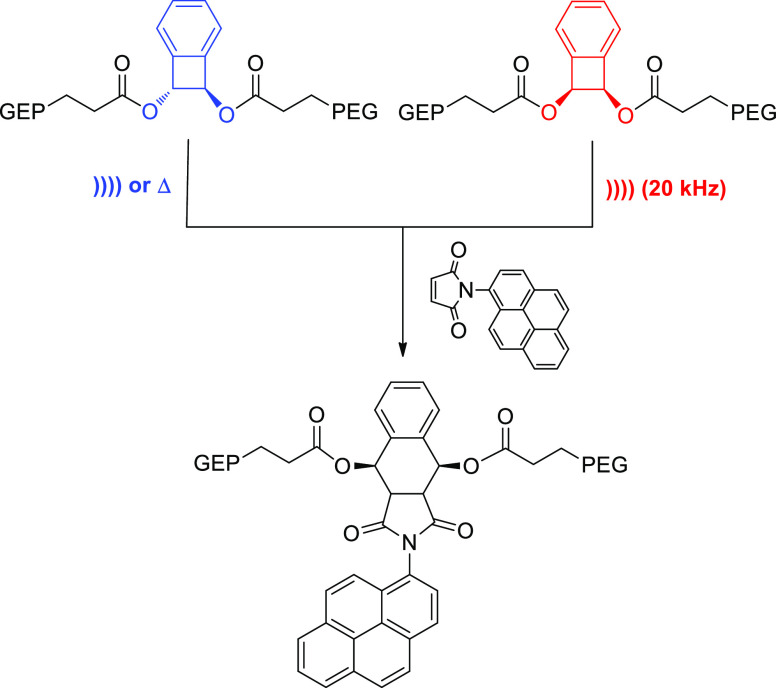
Forbidden Ring-Opening Reaction of Benzocyclobutene Mechanophores Induced by Ultrasonic Activation

*Divergent sonochemistry*, by which irradiation switches the reaction outcome to products unavailable under silent conditions, is widely documented, although surely the most prominent results have been reported in recent years in the context of *ultrasound as mechanical force*.^53−56^ Most examples involve polymer chains in solution irradiated by low-frequency probes in high-intensity fields for which mechanical effects and acoustic streaming are prevalent over chemical production of reactive species.^57,58^ As discussed above, the heterogeneous sonochemistry of small molecules will always be influenced by mechanical energy transfer as the turbulent implosion leads to friction and deformation at boundary surfaces.^59^ This tribochemical consideration mirrors the mechanical interpretation of ultrasonic agitation in liquids as an elongational flow field, where the strain rate depends on the time passed since the onset of cavitational collapse, the radius of the imploding bubble, and on the distance to the bubble.^60^ In homogeneous and/or dilute systems, however, the tensile forces lack directionality and molecular control is much more difficult to achieve than in standard mechanochemistry involving solid–solid and solid–liquid contacts.^61^ A way to convey mechanical activation to small molecules relies on the use of covalently linked polymer chains that, playing the role of large tweezers, concentrate and propagate the mechanical input on labile functional groups. Such entities now called *mechanophores* are embedded in or near the middle of polymer chains, thereby undergoing specific cleavage, whereas that mechanical activation fails if chains are not attached to both ends of the mechanophore. A salient result that challenges the validity of Woodward–Hoffmann rules for pericyclic reactions was described by Moore and associates on the sonication of a benzocyclobutene mechanophore in the middle of polyethylene glycol side chains (PEG) (Scheme 10).^62^ Both *cis*- and *trans*-derivatives experience ring opening affording an (*E,E*)-configured diene, whose presence could be corroborated by spectroscopic identification using a chromophore-containing molecule. Under thermal activation only the *trans*-isomer is expected to give the (*E,Z*)-diene.

It is obvious that mechanical scission caused by strain after bubble collapse is most likely responsible for polymer cleavage. Ultrasound does seemingly induce *conrotatory* opening in *trans*-benzocyclobutene and *disrotatory* motion in the *cis*-isomer. In principle, the conversion between (*E,Z*) and (*E,E*) products could involve a biradical transition structure; however, radical production appears to have a negligible effect in the present case. Accordingly, the intuitive conclusion is that thermally forbidden mechanisms may become *mechanochemically allowed pathways within a certain range of forces*. Theoretical simulations indicate that mechanical force can bias a given reaction toward a nonconventional fragmentation easier to achieve.^63,64^ Sonicated polymer solutions can mimic the response of macromolecules to mechanical loads in compressed solid polymers, although thermodynamically controlled mechanochemical reactions are difficult to assess in solution, as the equilibrium is re-established once the polymer escapes the elongational flow.^61^ Thermodynamically forbidden reactions, such as *cis*-to-*trans* isomerization of rigid bonds, can reach a lower transition barrier under mechanical strain that can be rationalized in terms of force distribution along a flexible polymer backbone.^65,66^

**Scheme 11:**
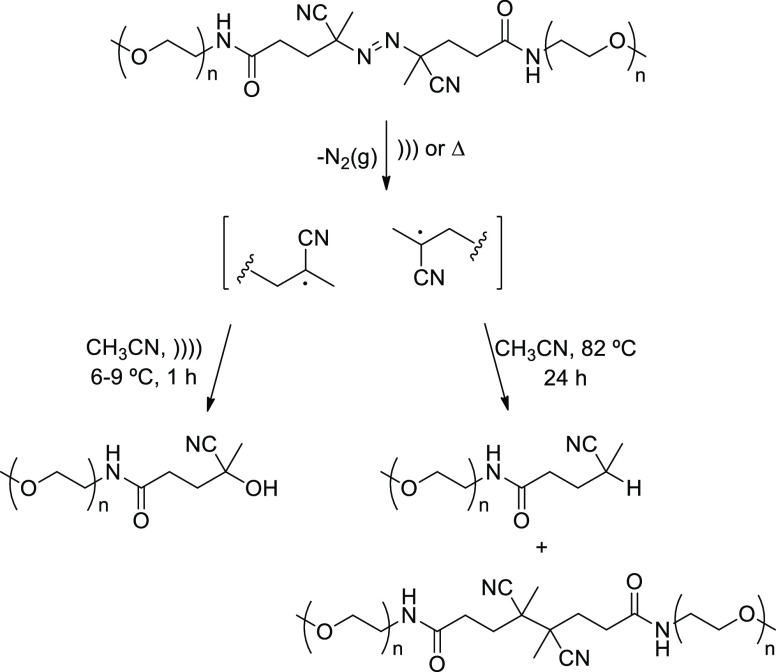
Divergent Sonochemical and Thermal Scission of Azobisnitriles within Long Polymer Chains

Although mechanisms and conditions for both polymer fragmentation and polymer formation in acoustic fields are well established and receive increasing attention,^67^ it is fair to say that the mechanophore concept grew up from the scission of a diazo bond stabilized by adjacent cyano groups and within two poly(ethylene glycol) chains.^68^ The divergent results under thermal and ultrasonic rupture are shown in Scheme 11.

Mechanical activation conducted with a high-intensity 20 kHz probe at mild temperature (6–9 °C) led in less than 60 min to selective formation of a cyano alcohol derivative, arising likely from oxidation of radical intermediates, which should also be generated by thermal fragmentation of the starting polymer. Thermal activation, however, required a higher temperature (82 °C) for 24 h and gave rise to products resulting from hydrogen abstraction and dimerization. The distinctive hydroxyalkylation under sonication evidence the role played by species produced after cavitational implosion, which are inaccessible by thermal degradation. This had already been observed by Nakamura et al. in a series of studies on sonochemical initiation of radical chain reactions where hydroxy groups are added to C–C double bonds.^69^

**Scheme 12:**
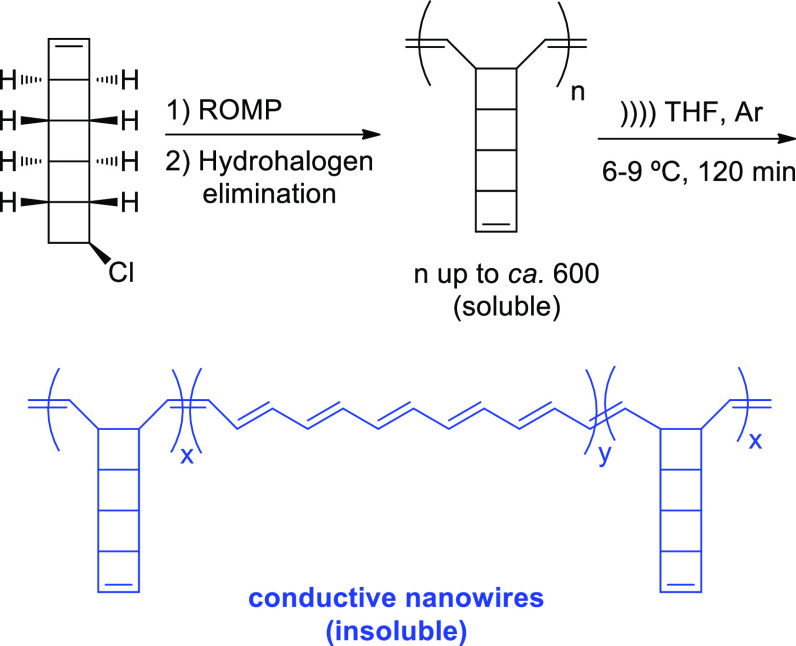
Formation of Polyladderene-Polyacetylene Copolymers under the Mechanical Action of Ultrasound

Numerous mechanophores and metallo-mechanophores undergo expeditious scission or transformation in the middle of polymer chains subjected to pulling forces caused by cavitation.^70^ An interesting and recent addition is the mechanochemical unzipping of polyladderenes, for which force causes *an extensive rearrangement* of the structure converting the nonconjugated and, hence, insulating, polyladderene structure into a conjugated polyacetylene with semiconducting properties.^71,72^ Due to the strained arrangement of fused four-membered rings, ladderenes undergo facile ring fragmentation. A terminal cyclobutene ring of the chloro-substituted [5]ladderane monomer breaks through ring-opening metathesis polymerization (ROMP) with a Grubbs ruthenium carbene catalyst. Further dehydrohalogenation affords homopolymers loaded with ladderene mechanophores along the skeleton. Sonication of the soluble mixture under argon causes extensive formation of a polyacetylene, which self-aggregates after prolonged irradiation resulting in an insoluble blue-colored polymer (Scheme 12).

**Scheme 13:**
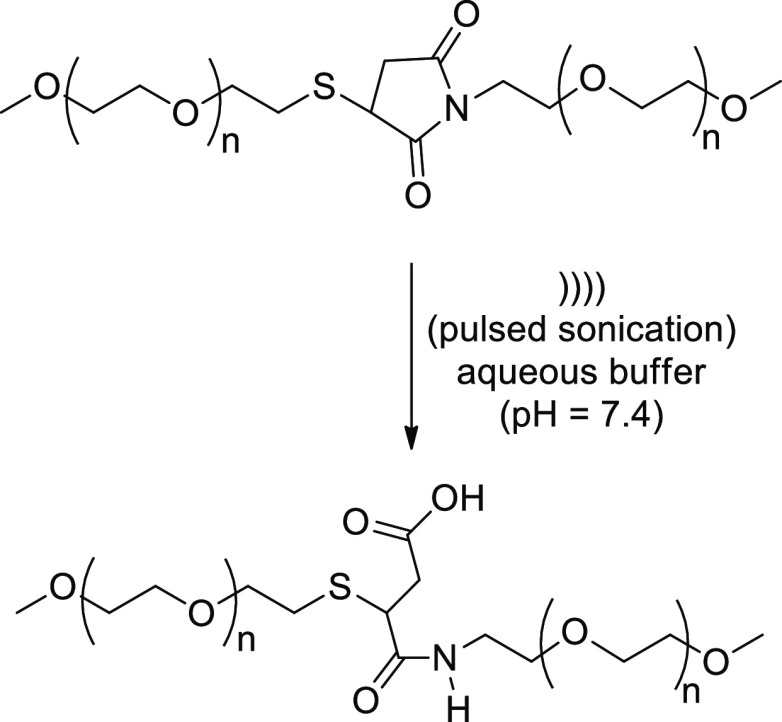
Mechanochemical Stabilization and Hydrolytic Acceleration of Maleimide–Thiol Adducts during Sonication

Application to bio-/physiological processes along with drug release represent extensions of sonolytically activated mechanophores. It is noteworthy a paradoxical example where mild *sonication stabilizes through stretching, compounds that would be unstable in the absence of force*, as reported recently for maleimide–thiol adducts.^73^ Such derivatives are employed in bioconjugation, although they are susceptible to retro-Michael reaction and, as a result, thiol exchange *in vivo* that diminishes the therapeutic effect of bioconjugates. Pulsed sonication in aqueous buffer at pH 7.4 of a polyethylene glycol (PEG)–maleimide reagent modified with trastuzumab, a clinically used antibody, accelerates adduct hydrolysis, thereby stabilizing the resulting conjugates and cutting significantly the competing retro-Michael reaction (Scheme 13). In addition, sonication does not change the binding of the antibody to its target ligand. In fact, antibody–PEG conjugates prepared under ultrasonic irradiation showed ligand-binding activity similar to those of silent conditions in an enzyme-based assay.

**Scheme 14:**
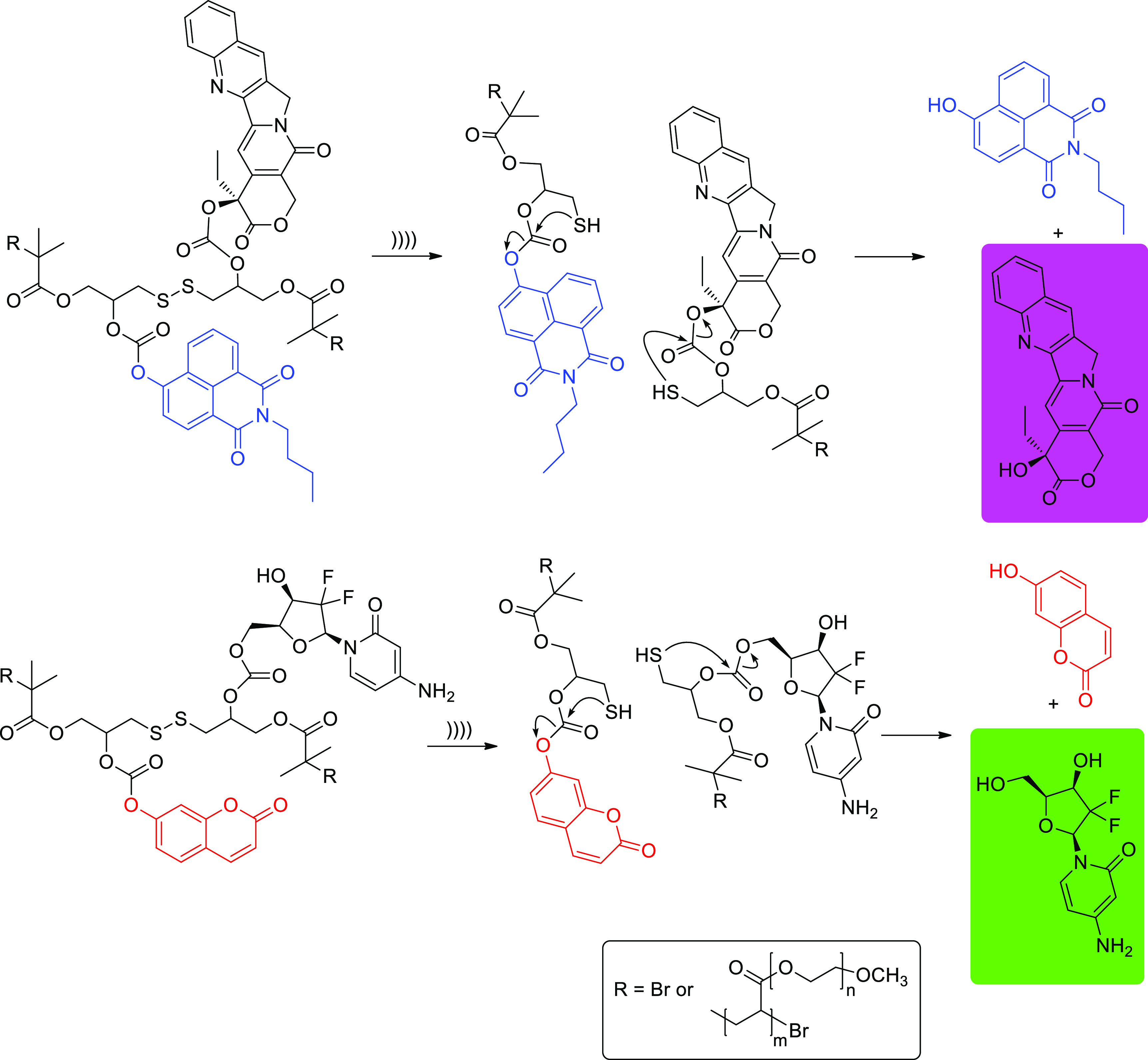
Mechanical and Reductive Cleavage of Disulfide-Centered Polymers Aimed at Drug Release by Ultrasonication

Likewise, mechanical scission of disulfides bearing cargo molecules within the center of a water-soluble polymer enables the liberation of anticancer molecules, such as camptothecin and gemcitabine, accompanied by fluorescent reporters.^74^ Sonication (20 kHz probe, 15.84 W/cm^2^) causes the reductive cleavage of the disulfide bond, and the resulting thiols are then involved in a subsequent 5-*exo-trig* cyclization that liberates the drug molecules (Scheme 14). As expected, terminally functionalized polymers failed to release the small molecules, thus confirming the stretching force of long chains on the centered mechanophore. Similar strategies have been tailored to drugs linked to polymers by covalent or noncovalent interactions. Thus, the aminoglycoside antibiotics neomycin B and paromomycin are released from RNA aptamers, as the carrier, by ultrasound-induced cleavage of both noncovalent interactions and labile covalent bonds with the phosphodiester RNA moiety. H-bonding interactions between a peptide and the antibiotic vancomycin (yet bound to a polymer or gold nanoparticle) can easily be disrupted by sonication.^75^ Complementary methods against infectious diseases combine both antibiotics, not necessarily linked to carriers, and the sonobactericide effect of bubble cavitation that fragments the cell wall and degrades other biomolecules required for bacterial function.^76^ Furthermore, cell membrane permeabilization, a process described as *sonoporation*, can be induced by stable cavitation at higher frequencies and enhances intracellular delivery of some drugs in tumor cells.^77^

At this stage, most convergent sonochemical reactions have traditionally been gathered within the classification of *false sonochemistry*, benefiting from mechanical action and enhanced mass transfer. However, they can be divergent in nature, as the nonirradiated reaction does not occur at all or requires conditions that can be circumvented by sonication. An interesting example is the ultrasonic treatment of silanes opening the path to new silicon structures. Due to the inverse polarization of the Si–H bond, small silicon hydrides are more reactive and energetic than the corresponding alkanes, and ignite in air spontaneously. Heating of silanes leads to pyrolytic disproportionation and oligomerization reactions producing long-chain silanes, but elevated temperatures (∼400 °C) and inert atmospheres are necessary. An alternative ultrasonic ring-opening polymerization of cyclopentasilane (Si_5_H_10_) for instance, causes ultrafast decomposition leading to silicon nanoparticles that can further be polymerized with ultrasound or UV light to Si-based materials for electronic or solar applications.^78^ The initiation of polymerization, assumed to be homolytic pyrolysis, is sonocatalytic in nature and not thermic, owing to the low macroscopic temperature of the solution (between 20 and 75 °C).

**Scheme 15:**
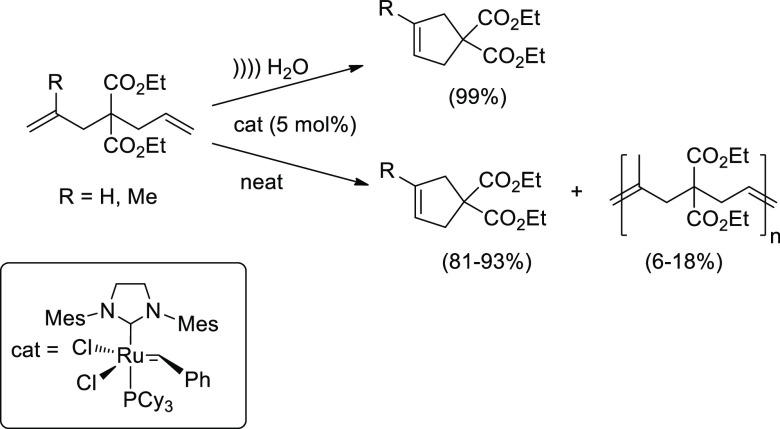
Ring-Closing Alkene Metathesis Using Acoustic Emulsification

Heterogeneous and aqueous biphasic reactions, for which ultrasound often gives rise to remarkable improvements without altering the conventional mechanism, have become appealing in the context of a green chemistry agenda.^5,6,79^ Enabling technologies make it possible reactions hitherto unknown having pluses in terms of higher yields and selectivities in shorter times. A critical appraisal based on green metrics should be advocated nevertheless.^80,81^ Enabling techniques, namely ultrasound, microwaves, electrochemistry or mechanochemistry, *are not green on their own*. It is ironic to claim benign syntheses under such “green conditions” while ignoring the amount of volatile solvents needed for product isolation and purification, as well as the fate of waste generated. That said, ultrasound exerts a beneficial homogenizing effect that enables water and water–organic cosolvent mixtures to be used, along with “in-water” and “on-water” reactions that depend strongly on interfacial contact.^82^ Early work by Ando and co-workers showed that ultrasound alone may replace the role of water and heterogeneous solid–solid and solid–liquid reactions are more favored than those run in homogeneous solution. As example, a strong oxidant like KMnO_4_ oxidizes alkenes smoothly in aqueous solution depending on the pH of the solution, but attacks double bonds very slowly in apolar solvents. The latter is much more efficient under sonication, presumably by destroying or altering the crystal lattice of the solid salt.^83^ Kinetic studies conducted under ultrasonic irradiation provided further evidence on hydrophobic interactions, which are responsible for the acceleration of some reactions in aqueous media. Sonication caused retardation of polar reactions in water, whereas rates were restored and enhanced in water–ethanol mixtures, likely associated with rapid micromixing of the reaction partners.^84^ As a consequence, sonication may replace the assistance of phase-transfer catalysis and facilitate the coupling of insoluble substrates in aqueous media. Representative developments that illustrate the efficient sonomixing of biphasic solutions are portrayed by olefin metathesis in aqueous emulsions or on-water reactions. A sonochemical ring-closing metathesis (RCM) at room temperature, without surfactants or organic cosolvents, takes place inside the water-insoluble droplets of the diene, leading to carbocycles in almost quantitative yields (Scheme 15).^85^ The ring-forming process occurs under neat conditions as well, although accompanied by side products resulting from diene oligomerization. The latter was prevented under the micellar conditions attained with sonication.

**Scheme 16:**
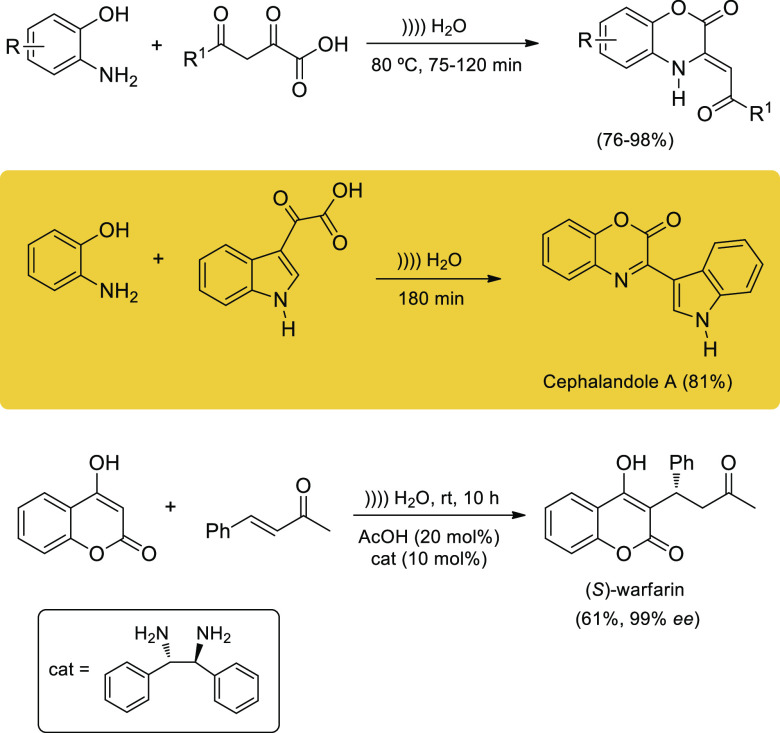
Sonochemical Syntheses of Heterocyclic Drugs in Aqueous Media

A one-pot synthesis of the antitumoral indole alkaloid cephalandole A involves the catalyst-free condensation of 2-aminophenol with an indolyl carboxylic acid derivative in water under ultrasonic agitation. This application discloses a convenient atom-economical protocol for the construction of 2-oxo-benzo[1,4]oxazines in aqueous media that combines ultrasound and heating.^86^ A related transformation, using a chiral diamino ligand as catalyst, (*S,S*)-diphenylethylenediamine, affords the anticoagulant drug warfarin in optically pure form^87^ and could be accomplished in sonicated aqueous solution (Scheme 16).

**Scheme 17:**
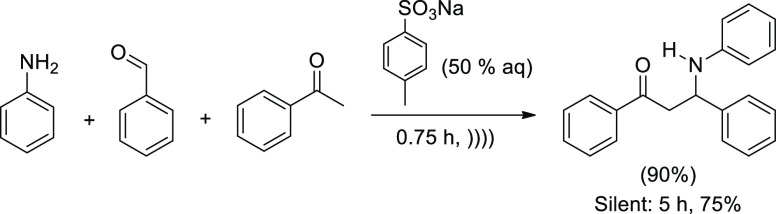
Synthesis of β-Amino Carbonyl Compounds by Three-Component Reaction in Hydrotropic Solution Irradiated with Ultrasound

The use of surfactants can increase the solubility of organic substrates and have a positive effect on inertial cavitation, in part due to the enhanced growth rate of the bubbles.^88^ A cationic surfactant, cetyltrimethylammonium hydroxide, has been used to increase the solubility of isatin and aryl/heteroaryl ketones, whose condensation gives rise to quinolines in 75–95% yield at room temperature under sonication (22 kHz, 40% amplitude). Irradiation increases the reaction rate (by *ca*. 3-fold) and avoids strong bases like NaOH or KOH employed in the silent reaction. It is pertinent to note, in terms of energy savings (energy supplied in kJ per mass of product), that sonication saved more than 78% energy with respect to the nonirradiated procedure.^89^ A similar effect to that of surfactants on solvent’s surface tension can be obtained by *hydrotropes* in aqueous solution. The term hydrotrope denotes a nonmicelle forming substance, i.e., compounds that do not have a critical concentration above which self-aggregation takes place, while increasing the solubility of hydrophobic compounds. Aqueous hydrotropic solutions merge both low vapor pressure and increased viscosity, relative to pure water, thus causing stronger cavitational collapse during compressional cycles and therefore faster reactions. A hydrotropic solution containing sodium *p*-toluensulfonate at a concentration (50% w/v) that allows the maximum solubilization of acetophenones, aniline, and aryl aldehydes facilitates the three-component coupling when sonication is applied (Scheme 17).^90^

## Cavitational Effects Can Be Used to Resolve Mechanistic Pathways

**Scheme 18:**
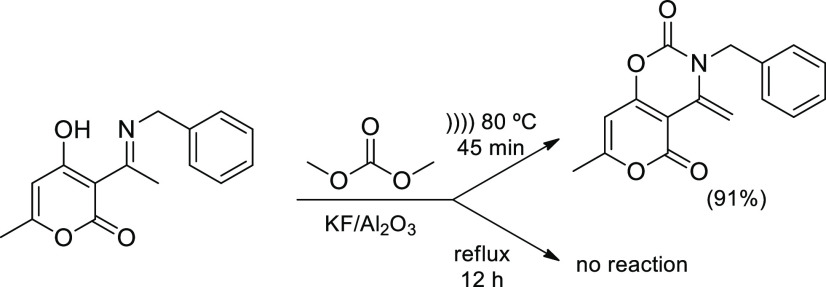
Mismatched Cavitational and Thermal Effects on the Kinetic Activation of a Solid Catalyst

The late Nobelist George Olah once said that “mechanisms cannot be proven, only disproven”. By determining the influence of acoustical parameters on rates and reaction products, a sonochemical study can inform on the actual effects of cavitation. As witnessed in the preceding results, ultrasonic activation is of enormous value in chemical processing, but investigation in sonochemical mechanisms is rather unusual. Seeing as a reaction does not occur under silent conditions, cavitation points to effects other than thermal activation. In a high atom-economy construction of fused heterocycles, pyrano[3,4-*e*][1,3]oxazines, extruding 2 equiv of methanol only (Scheme 18), reagents were mixed on KF/alumina as catalyst and sonicated (37 kHz), giving rise to the product after 45 min at 70–80 °C.^91^ No reaction occurred using the same catalyst at the same temperature in the absence of irradiation, even after 12 h. Given the low volatility of substrates, they will not undergo pyrolytic cleavage in the bubble and cavitation just provides sufficient kinetic energy to surmount the reaction barrier. At such a frequency and low power, the kinetic effect might stem primarily from acoustic streaming. However, the thermal effect alone has little effect on the catalyst’s activation. The key role of cavitation should instead be related to shockwaves and microjets that cause local deformation at the solid surface and increase the possible reaction sites.

Nearly two decades ago, we coped with the interpretation of a sonochemical cycloaddition, which are still among the most challenging cases to disentangle. Both moderate accelerations and greater yields have been reported for a few [4 + 2] and [3 + 2] cycloadditions, the most successful involving quinones as dienophiles as well as the use of azadienes.^92^ The redox properties of such partners might then lead to an electron transfer instigated by ultrasound giving a radical cation generated by the diene or its dienophile. This situation resembles the mechanism of radical cycloadditions and related pericyclic reactions developed by Bauld and associates, though initiated by aminium radical cations.^93^

**Scheme 19:**
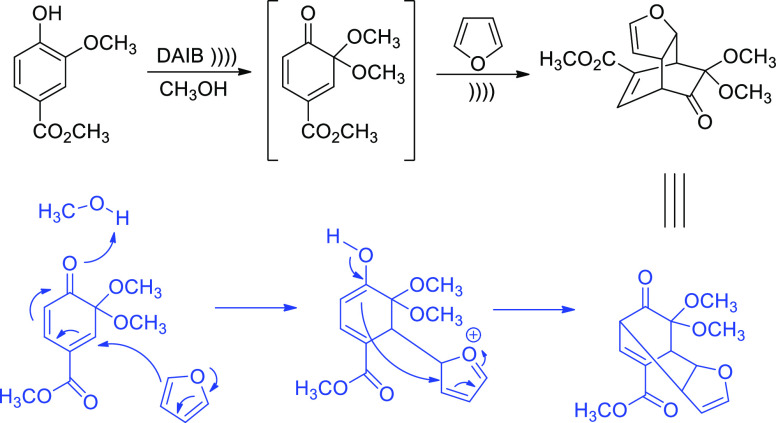
Addition of Furan to Masked *o*-Quinones Proceeding through a Probable Stepwise Mechanism

**Figure 3:**
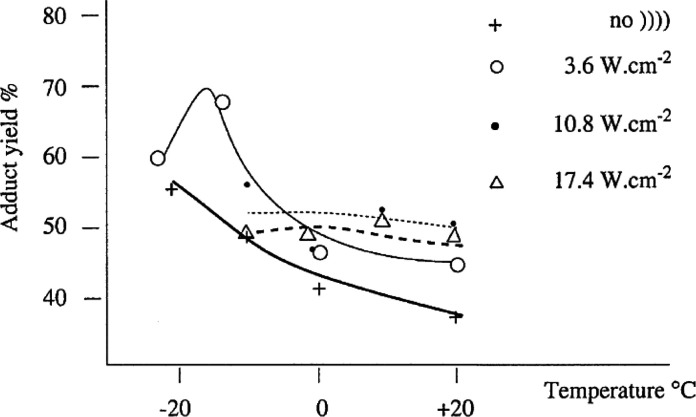
Cycloadduct yield as function of temperature at three acoustic powers for the reaction of methyl vanillate and furan. Reproduced with permission from ref (94a). Copyright 2003 Elsevier B.V.

The addition of furan (as 2π component) to masked *o*-benzoquinones, generated *in situ* by oxidation of the corresponding phenol with (diacetoxyiodo)benzene (DAIB), was accelerated by sonication, though neither the regiochemistry nor diastereoselection were affected (Scheme 19).^94^ Yields were dependent on acoustic energy, temperature, and solvent composition. A radical initiator (aminium salt) had no appreciable effect on the yield relative to the silent process, nor did oxygen/argon atmospheres alter the extent of cycloadduct formation. The combined effect of acoustic power and temperature is noticeable (Figure 3). Working with a 30 kHz probe between −20 and +20 °C at three different energy levels (3.6, 10.8, and 17.4 W/cm^2^), all above the cavitational threshold, the yields were the lowest at the highest power and remained practically constant as temperature varied. At 10.8 W/cm^2^, the yields improved slightly, whereas higher yields were attained at the lowest acoustic power (3.6 W/cm^2^) with a significant variation with temperature (up to 70% at −10 °C). The paradoxical anti-Arrhenius effect of sonochemical reaction was evident as cavitational collapse enhanced by lowering the temperature and hence the solvent’s volatility. The increase in viscosity as temperature decreased halted the cavitational activation and the cycloaddition slowed down at −20 °C. All such results are inconsistent with a concerted process while supporting a stepwise double Michael addition as shown in Scheme 19. The formal cycloaddition could also be accelerated under Lewis acid catalysis and sonication at −10 °C. Given the homogeneous working conditions, the question is whether there is any sonochemical effect not related to a mechanistic switching. UV–vis monitoring in the presence and absence of furan revealed that sonication greatly increases the dispersion of the parent quinone, thus inducing an efficient mixing in the system without mechanical stirring. The sonochemical batch reactor behaves, at least for small volumes, like a flowlike reactor where convective currents avoid local supersaturation and accounts for the observed improvements.

Epoxidation and oxidations not involving radical pathways appear to be candidates to elucidate the effect of sonication as changes in frequency and intensity will produce different amounts of oxidizing free radicals. The use of aqueous hydrogen peroxide for epoxidation of hydrophobic alkenes needs the assistance of cosolvents and phase-transfer surfactants.^95^ A fine example of mechanistic switching, yet giving the same product, takes place by combining ultrasound and a hydrophobic ionic liquid, instead of acetonitrile under silent conditions, as reported for the epoxidation of alkenes with H_2_O_2_/NaHCO_3_ and a manganese–porphyrin catalyst.^96^ The bleaching of the catalyst in CH_3_CN favored an epoxidation route where peroxycarbonate (NaHCO_4_) was the actual oxidant. In contrast, the ionic liquid (methyloctylpyrrolidinium-NTf_2_) protected the catalyst from degradation and ultrasonic irradiation (20-kHz probe, *P*_elect_ = 11.5 W, 0.79 W/mL) kinetically induced the formation of high-valent oxo-Mn-porphyrin as oxidant. The ultrasonic effect could be unambiguously demonstrated by using a chiral bis-binaphthyl Mn-porphyrin as catalyst. Enantioenriched epoxides were obtained in the ionic liquid as solvent, while a racemate formed in CH_3_CN without sonication owing to decomposition of the metalloporphyrin.

The same team conducted epoxidation of *cis*-cyclooctene at much higher frequency (800 kHz) using H_2_O_2_ and H_2_WO_4_ as catalyst.^97^ Tungstate salts are in fact appropriate and sustainable catalysts in the sonochemical processing of biomass derivatives.^98^ At high frequency, the increasing production of OH radicals coming from either water or H_2_O_2_ sonolysis would favor a more efficient and faster path not accessible without irradiation. Under conventional conditions and due to the strong exothermicity of epoxidation, there is a sharp increase of temperature (from 25 to 95 °C in 5 min) that renders the process uncontrolled. To check the influence of sonochemical parameters, high-frequency irradiation was applied at low acoustic power to ensure a steady 60 °C temperature, giving rise to a retarded reaction with respect to silent conditions. Surprisingly, little H_2_O_2_ (less than 2%) is decomposed by ultrasound as inferred from measurements with a chemical dosimeter, and higher loadings of tungstic acid do not catalyze hydrogen peroxide decomposition either.

**Scheme 20:**
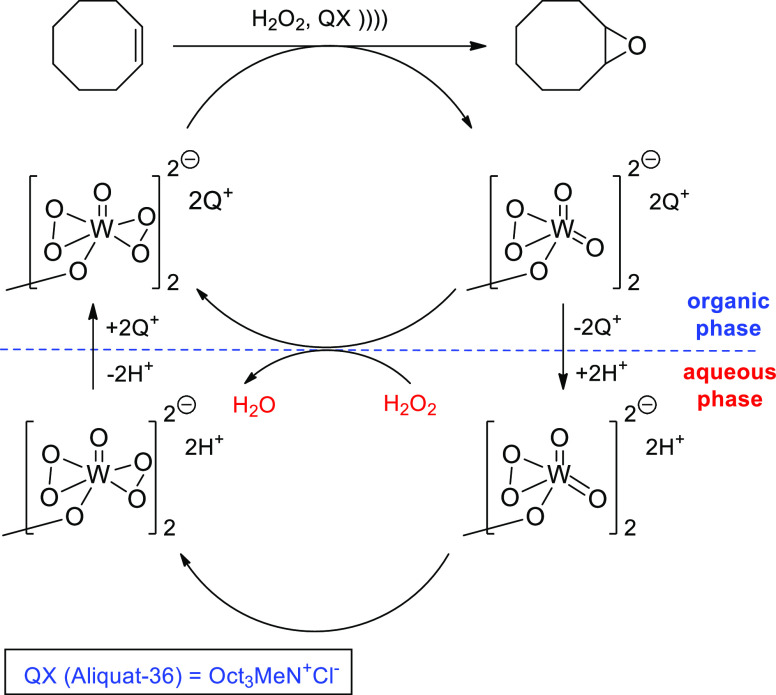
Catalytic Cycle Induced by High-Frequency Ultrasound in the H_2_O_2_-Mediated Biphasic Epoxidation of Cyclooctene

These results suggest that OH^•^ radicals formed by sonolysis do not undergo side reactions that decompose progressively H_2_O_2_. Rather, the sonochemical conditions favor the milder formation of stable peroxotungstate species. The use of biphasic conditions with an onium salt as PT catalyst was explored, but no effect from radical production could be determined. The pluses of sonication are related to an efficient mixing, in line with the above-discussed cycloaddition, and enhanced phase transfer to bring active peroxotungstate species from aqueous to organic phase to epoxidize *cis*-cyclooctene (Scheme 20). While the silent reaction produces higher conversion and yield after 15 min, almost complete selectivity toward epoxide formation is provided by sonication between 15 and 45 min. This result is linked to the accurate control of temperature (between 60 and 80 °C), which also allows a low loading of H_2_WO_4_ as catalyst compared with the nonirradiated reaction.^97^

**Scheme 21:**
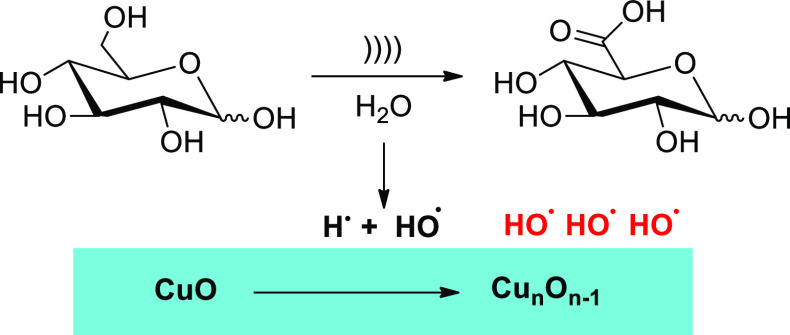
Selective Sonocatalytic Oxidation of d-Glucose to d-Glucuronic Acid under High-Frequency Ultrasound

A more direct participation of water sonolysis at high frequency that alters product selectivity is evidenced by glucose oxidation catalyzed by cupric oxide at 550 kHz.^99^ While ultrasound alone and Cu(II) catalysts can independently oxidize glucose to gluconic acid, water sonolysis at high frequency under argon leads to H^•^ and OH^•^ radicals, the former being trapped by the oxygenated lattice of the catalyst. This leaves concomitantly a broader coverage of OH^•^ radicals on the CuO surface that selectively oxidizes glucose to glucuronic acid (Scheme 21). Moreover, cavitation bubbles are much smaller at high frequency than at the usual 20 kHz, thus preventing the mechanical damage of shock waves on the catalyst after implosion. Gluconic acid is formed as byproduct in low yield (<10%), probably due to diffusion of radicals into the bulk liquid. The use of a radical scavenger inhibited the formation of glucuronic acid, thus illustrating the synergistic effect of OH^•^ radicals and the solid catalyst.

## Piezoelectricity and Piezo-Redox Chemistry

**Figure 4:**
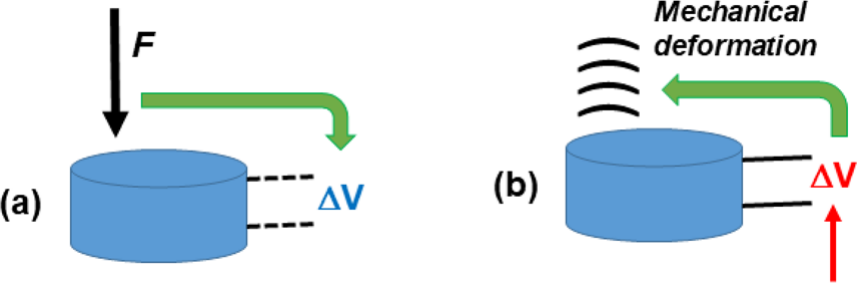
Schematic representation of direct (left) and inverse (right) piezoelectric effects.

Ultrasonic energy is transmitted by power transducers, which have the ability to convert high-frequency electrical fields into mechanical vibration. This takes advantage of the *piezoelectric effect* in certain crystals, by which a mechanical force applied to an ionic crystal produces an electric polarization, and therefore a measurable voltage. This is the *direct* piezoelectric effect. The *inverse* piezoelectric effect occurs when a voltage applied to the material leads to mechanical strain (Figure 4). Piezoelectricity has the origin in the lack of a center of symmetry in the crystal structure and the anisotropic effect can only be observed in some directions of natural and artificial crystals.^100^ Along with piezoelectric transducers, magnetostrictive materials have been employed as well, where a magnetic field converts into mechanical motion. However, the development of piezoelectric ceramics with improved mechanical properties make these materials suitable for most power ultrasonic systems. Furthermore, piezoelectric crystals can also be ferroelectric with a hysteresis loop, a nonlinear relationship between polarization and the applied electric field.^101^ Common piezoelectric materials include oxides like BaTiO_3_, PbTiO_3_ and LiNbO_3_ together with more complex compositions, albeit in all cases they show perovskite-type structures. It is noteworthy that piezoelectricity can be substantially improved by metal doping, which may find further applicability in sonar and ultrasonic imaging.^102^

Piezoelectric materials, in the form of massive solids or particles, have shown to catalyze redox reactions under mechanical force, like those produced by shear conditions of low-frequency ultrasound as well as strong mechano-vibration. It is believed that charges occur in the catalyst, thereby generating a strain-induced voltage that can be coupled with electron transfer with substrates. Such *mechanoredox* catalysts can be viewed as analogous to photoredox catalysts, which hold significant promises for synthetic explorations. In order to understand how piezoelectricity can mediate a redox transformation, some physical considerations are needed, the basis being that aligned dipoles exist within the solid. A compressive force will decrease the dipole separation, thus leading to a net charge on the faces of the material and hence a voltage. Conversely, a tensile force will produce an opposite voltage. As a result, a piezoelectric material combines both mechanical and electric properties.^103^ For an elastic solid, the relation between stress (σ) and strain (ε) is given by Hooke’s law in the form
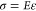
3where *E* is Young’s modulus. For the remaining discussion, however, and given the potential confusion between *E* for Young’s modulus and the standard nomenclature for electric field strength (with identical symbol), Hooke’s law should now be expressed as
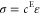
4where *c*^E^ is the elastic Young’s modulus and the superscript (E) denotes the electrical conditions under which the modulus is measured, in this case, a constant electric field. On the other hand, the behavior of a capacitor can simply be described by
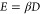
5with *E* being the electric field, *D* the electric displacement and β the dielectric coefficient. In a piezoelectric material σ and *E* are coupled in the way that application of force not only induces mechanical stress, but also produces electric charge. In mathematical form, the following equations dictate the behavior through a *piezoelectric constant* (*h*) that links the mechanical and electrical responses:
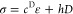
6
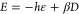
7the only variation being that the *c*^D^ modulus replaces the above *c*^E^ to indicate measurement under constant charge conditions.

**Figure 5:**
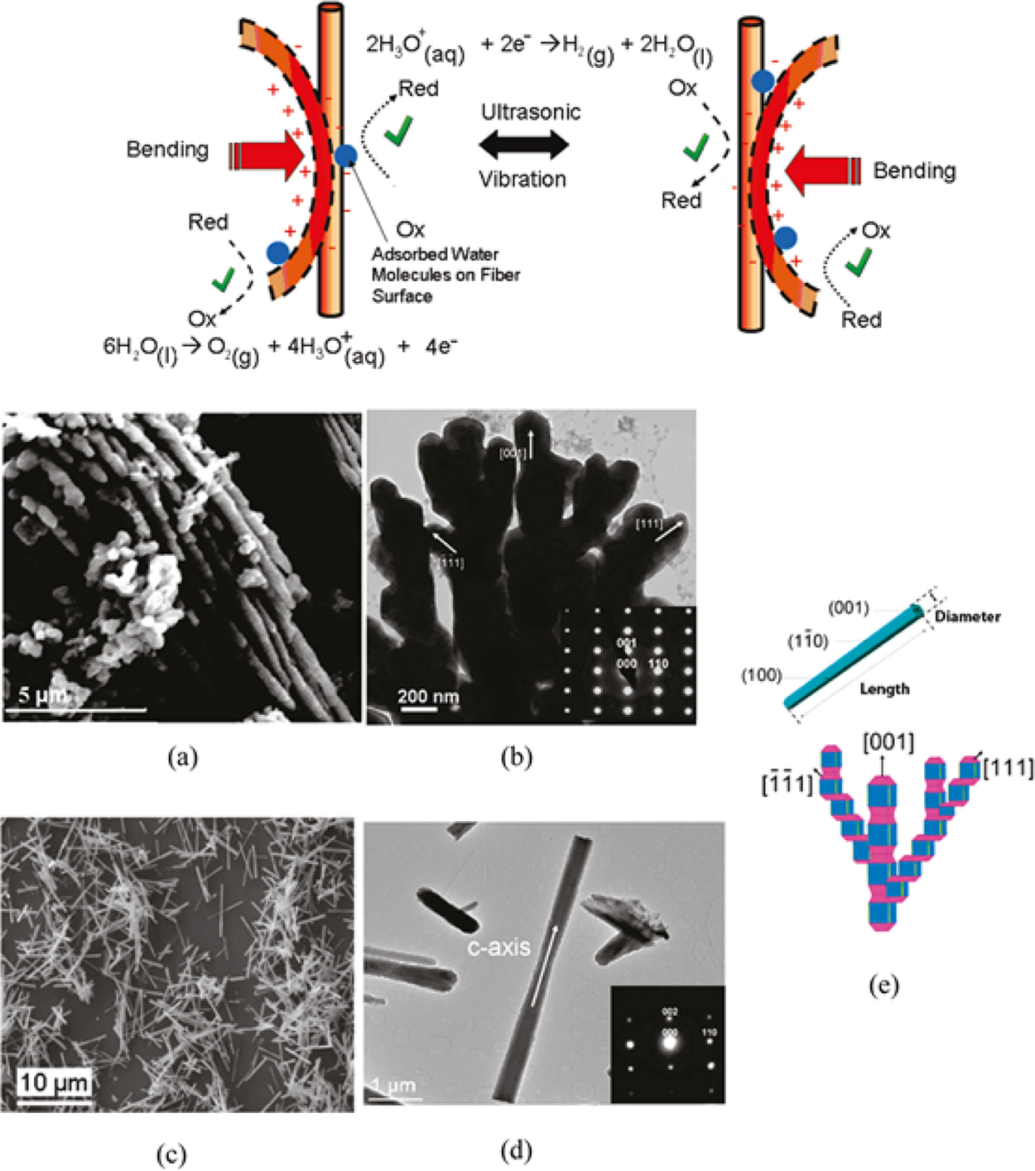
(Top) Micrometer-sized BaTiO_3_ or ZnO experience acoustic vibrations that cause water splitting via piezoelectric effect. (Bottom) (a) SEM and (b) TEM images of BaTiO_3_ dendrites grown on a glass substrate; (c) SEM and (d) TEM images of ZnO fibers grown on a Si(100) wafer. (e) Morphologies of a single ZnO fiber (upper image) and BaTiO_3_ dendrite (lower image). Reproduced from ref (104). Copyright 2010 American Chemical Society.

Ultrasonic agitation of a piezoelectric material can be sufficient to generate the electrochemical potential required for water splitting.^104^ Thus, hydrothermally synthesized ZnO microfibers (also exhibiting piezoelectric properties) and BaTiO_3_ microdendrites were vibrated with ultrasound, thereby developing a strain-induced charge on their surfaces and causing the liberation of H_2_ and O_2_ from water (Figure 5). This phenomenon is conceptually different from other sonochemical and mechanocatalytic processes where the reagent undergoes a redox reaction to split water.^105,106^ The piezoelectric catalyst is involved in donating strain-induced electrons and holes without decomposition or redox change. Irradiation with an ultrasonic transducer (which actually harnesses the inverse piezoelectric effect) leads to the rapid production of H_2_ and O_2_, with gas generation ceasing when ultrasound is turned off. This kind of miniature devices for low-cost water splitting can be advantageous over other electrochemical and photochemical procedures, although optimum performance depends on both lengths and resonance frequency of the piezoelectric material. Longer fibers may undergo a greater bending and hence an increase in strain-induced voltage.

**Scheme 22:**
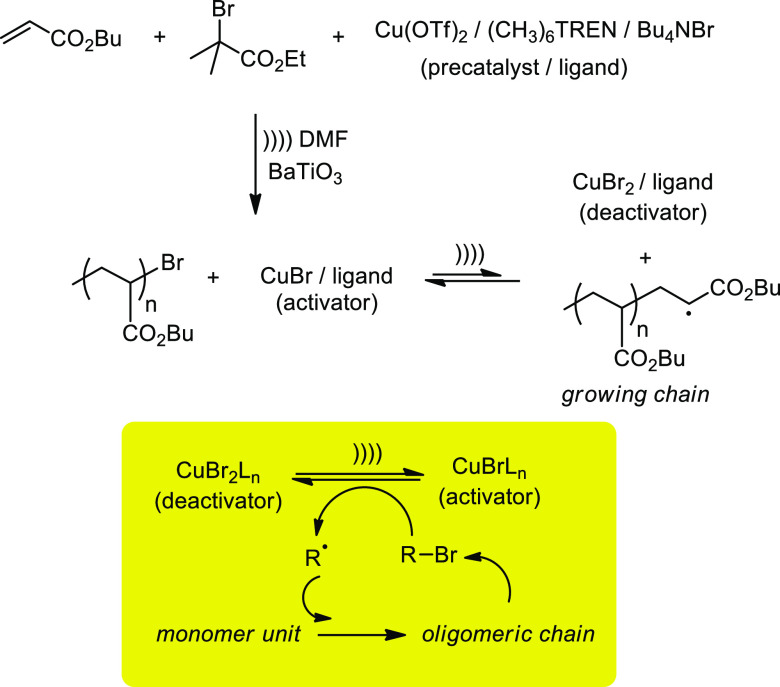
Sonication-Induced Mechano-Radical Polymerization with Piezoelectric BaTiO_3_. Bottom: Mechanistic Simplification of the Sonochemical Activation

Piezoelectric (nano)particles can be literally employed as *chemical transducers* for reduction of organic molecules or metallic precatalysts required for coupling reactions or radical polymerizations.^107^Scheme 22 depicts the radical polymerization of *n*-butyl acrylate using a suspension of BaTiO_3_ NPs sonicated in the presence of *n*-butyl acrylate, ethyl α-bromoisobutyrate as initiator, and equimolar amounts of Cu(OTf)_2_, Me_6_TREN, i.e., *N,N,N′,N′,N″,N″*-hexamethyl[tris(aminomethyl)amine], and Bu_4_NBr as the catalyst precursor.^108^ The piezoelectric material reduces the Cu(II) precatalyst to its active Cu(I) form that activates the alkyl halide. Ultrasound not only produces alkyl radicals by homolytic cleavage but also sufficient mechanical input to control polymer growth.

**Scheme 23:**
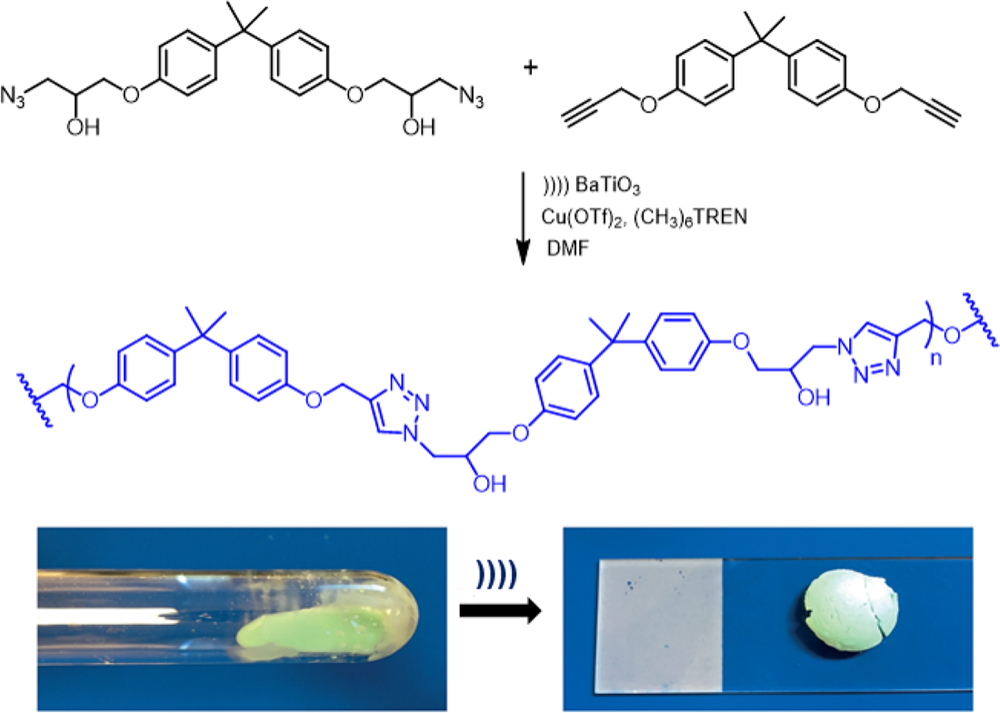
Click Azide–Alkyne Polymerization Using Cu(I) Species Generated by Ultrasonic Reduction of Cu(II) Precatalyst with BaTiO_3_ Particles Photo (bottom) reproduced with permission from ref (109). Copyright 2018 Wiley-VCH Verlag GmbH & Co.

Combination of BaTiO_3_-mediated step-growth polymerization and Cu-catalyzed azide–alkyne click cycloaddition using the above-mentioned Cu(II)-precatalyst gave rise to a linear polytriazole.^109^ Further application of this protocol to a polyurethane–trialkyne mixture afforded a polymer, which could be cross-linked mechanochemically. The resulting gel solidified after prolonged ultrasonic irradiation (Scheme 23).

**Scheme 24:**
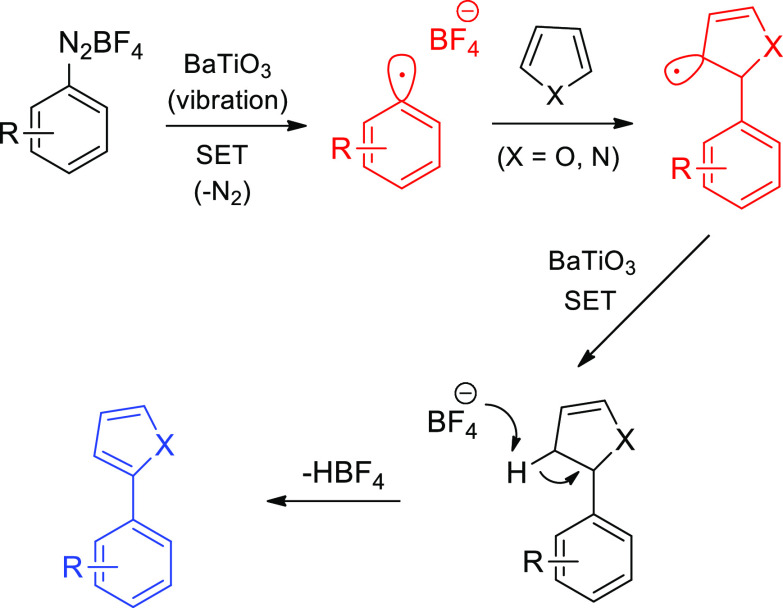
Mechanoredox Arylation Mediated by a SET Pathway with Piezoelectric Material

Mechanical activation other than ultrasound in the presence of piezoelectric crystals can trigger a similar mechano-redox reaction. Above a critical agitation threshold, highly polarized particles will be formed serving as strong reductants to transfer electrons to small molecules, followed by oxidative quenching of a donor molecule, thus leading to bond formation. This has been shown by Kubota and co-workers using ball milling to agitate BaTiO_3_ that reduces aryl diazonium salts, which are then applied to arylation and borylation reactions (Scheme 24).^110^ It is worth pointing out that ball milling generally provides sufficient stress to activate the piezoelectric material, while grinding or other mechanical methods afford poor or no results, as revealed by solvent-free C–H trifluoromethylation of *N*-heterocycles and short peptides developed by the same group. Moreover, the optimum performance hinges on the number and size of the balls and longer reaction times.^111^

**Figure 6:**
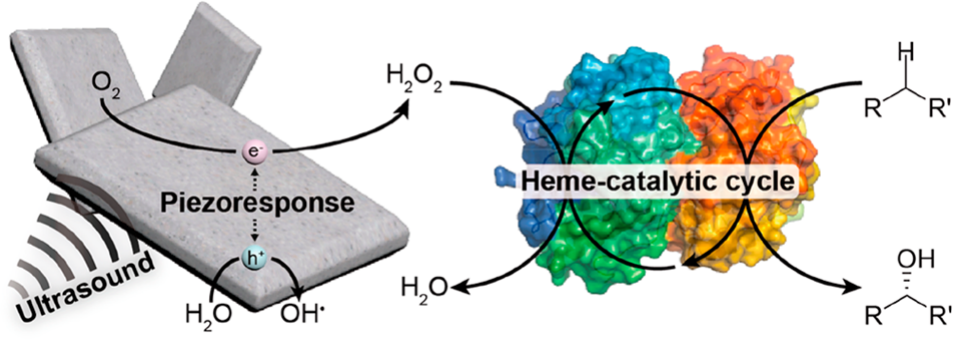
Sonication-induced catalytic C–H oxyfunctionalization using piezoelectric BiOCl microsheets plus an unspecific peroxygenase. Reproduced from ref (113). Copyright 2020 American Chemical Society.

Piezoactivation of biomolecules, like enzymes, can inspire major developments, although it is well-known that ultrasound affects enzyme activity and may cause extensive degradation after long irradiation times.^112^ In a recent study, Yoon et al. were able to implement a peroxygenase catalyzed oxyfunctionalization that exploits the piezocatalytic generation of H_2_O_2_ via oxygen reduction on bismuth oxychloride (BiOCl) under ultrasound (40 kHz, 70 W).^113^ Bismuth-based ceramics have gained popularity for transducer applications as improved lead-free ferroelectrics,^114^ even though bismuth salts remain underestimated as piezoelectric reagents^115^ compared to titanate compounds.^116^ An illustration of the oxyfunctionalization reaction is shown in Figure 6, where ultrasonic activation of BiOCl in aqueous buffer solution containing a recombinant unspecific peroxygenase from *Agrocybe aegerite* (300 nM) accelerated the selective hydroxylation of ethylbenzene to enantiopure (>99% ee) (*R*)-1-phenylethanol. The mechanism should involve the reaction of electrons from the piezoelectric material with O_2_ and H_2_O leading to superoxide (O_2_^•–^) and OH^•^ radical intermediates. The authors hypothesized that such radical species may inactivate peroxygenase, thus hampering its catalytic activity. To minimize the oxidative stress of short-lived OH^•^ radicals, the enzyme was spatially separated from BiOCl and placed in a dialysis membrane bag, which allowed the diffusion of H_2_O_2_ and products without altering the catalytic effect.

## Flow and Automation: The Present and Future

Chemistry from this century on should be dominated by the juxtaposition of automation and machine-learning algorithms, which will lead to (almost) complete robotization of synthetic and operational steps.^117,118^ The increasing adoption of continuous flow systems over batch reactors, still with timid implementation by the industry, has much to do with more efficient processes in terms of costs and risks, and in agreement with a more sustainable chemistry.

**Figure 7:**
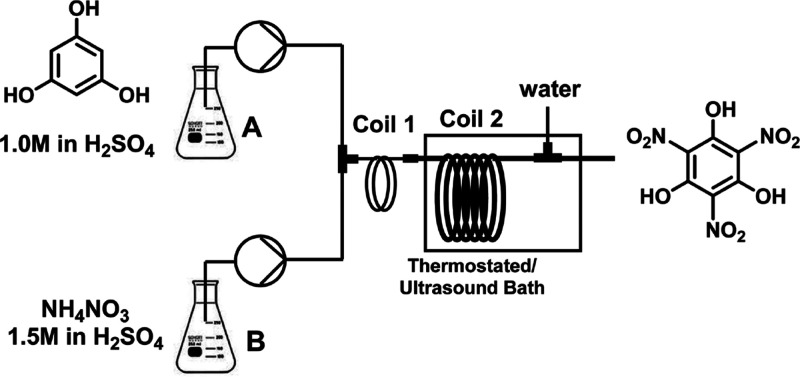
Continuous flow nitration of phloroglucinol assisted by sonication. Reproduced from ref (125). Copyright 2014 American Chemical Society.

While industrial applications of acoustic and hydrodynamic cavitation require scale-up considerations (reactor design for instance),^119,120^ which are not covered here, ultrasound and flow systems are ideal partners as one moves downward, i.e., *miniaturization*, because acoustic microfluidics constitutes an emerging technology enabling cell and particle manipulation.^121^ Moreover, bubble-based robotics and on-a-chip systems should help to develop novel acoustic micro/nanomotors and sensors.^122^ For the purpose of this Perspective, we are concerned with acoustoflow synthesis at the meso- and microscale levels. Unlike batch conditions, flow and microflow devices exhibit enhanced heat and mass transfers along with improved reaction kinetics, due to the small path occupied by reagents in channels of microscopic diameters, and enable the preparation of organic compounds, especially active pharmaceutical ingredients.^123^ In addition, the reduced dimensions allow the facile penetration of external energy souces, namely ultrasound, microwaves, or light.^124^ The rapid formation and consumption of intermediates and products, generated in small amounts, entail the safe handling of hazardous substances. Overall, such benefits are not only linked to greater efficiency, but also to lower environmental impact. The point is well illustrated by a continuous nitration of phloroglucinol leading to a potentially explosive polynitrophenol as shown in Figure 7.^125^ The nitration reaction requires an oxidizing and toxic mixture of sulfuric acid and ammonium nitrate, whose manipulation jeopardizes the protocol, even in dilute conditions. Mixtures of mono- and trinitrophloroglucinols are usually obtained, although the latter could be generated in 98% yield (determined by HPLC) at 40 °C using a thermostated ultrasonic bath, which homogenized the reaction mixture as well.

**Figure 8:**
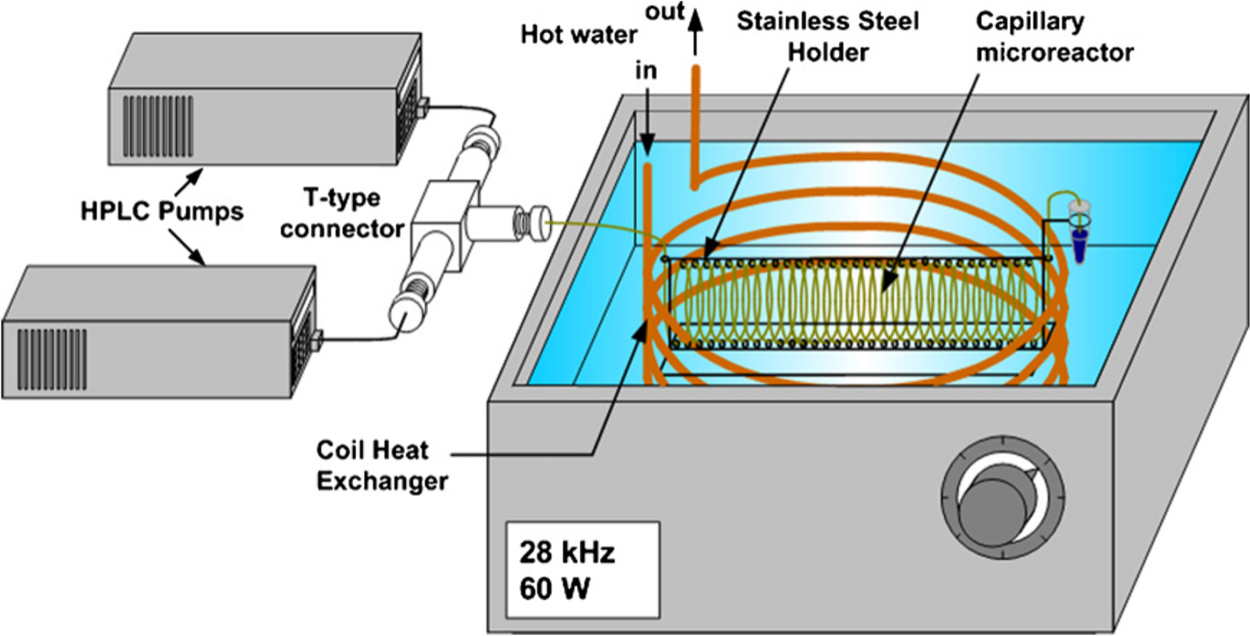
Capillary sonoreactor for phase-transfer catalysis. Reproduced with permission from ref (127). Copyright 2009 Elsevier B.V.

The most serious drawback of a microfluidic reactor is associated with clogging of small channels caused by heterogeneous mixtures and/or concomitant precipitation of products, which can be avoided by cavitational agitation.^126^ The best way to overcome that limitation is to immerse the microchannel device in an ultrasonic bath as depicted in Figure 8. The system in question was employed for the phase-transfer reaction of benzyl chloride and sodium sulfide catalyzed by an ammonium salt.^127^

**Figure 9:**
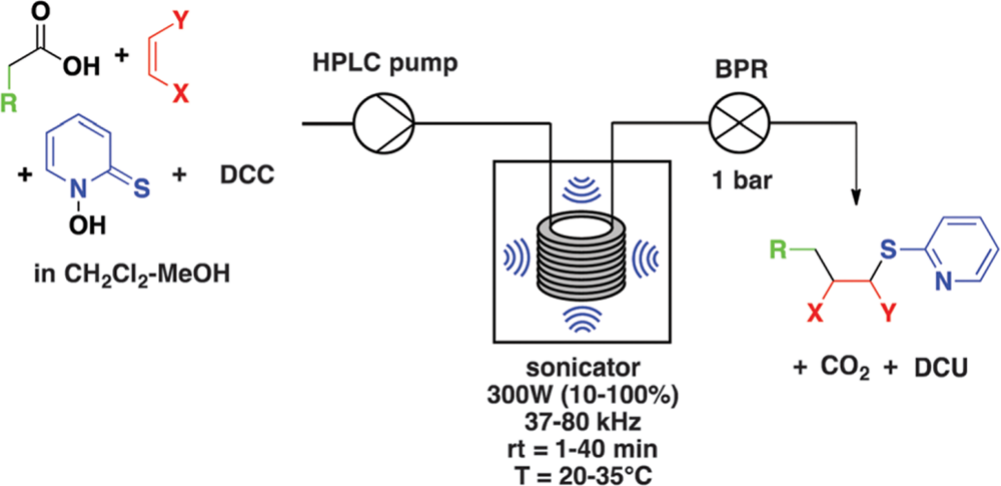
Sonochemical Barton decarboxylation of carboxylic acids under continuous flow. The protocol was optimized for hexanoic acid and *N*-phenylmaleimide using dicyclohexylcarbodiimide (DCC). Reproduced with permission from ref (128). Copyright 2016 the Royal Society of Chemistry.

An optimized Barton decarboxylation has been achieved under ultrasonic continuous flow, which enabled large-scale synthesis.^128^ The reductive decarboxylation of hexanoic acid was selected as a case study, using CH_2_Cl_2_–MeOH as solvent system pumped by HPLC. The mixture was entered into a tubular reactor (Teflon coil, 0.8 mm internal diameter and 40-m length) heated in an ultrasonic bath below 35 °C (Figure 9). Both power (10 to 100%, 300 W) and frequency (37 to 80 kHz) were regulated, while in order to ensure a liquid mixture the pressure was set to 0.1 MPa by a back-pressure valve (BPR). Being a radical mechanism, the Barton decarboxylation took advantage of both mechanical and chemical effects provided by cavitation, by bringing energy for radical production (H^•^ and OH^•^) and removing oxygen from the solution. The transformation proceeds with formation of CO_2_ and dicyclohexyl urea as byproducts.

**Figure 10:**
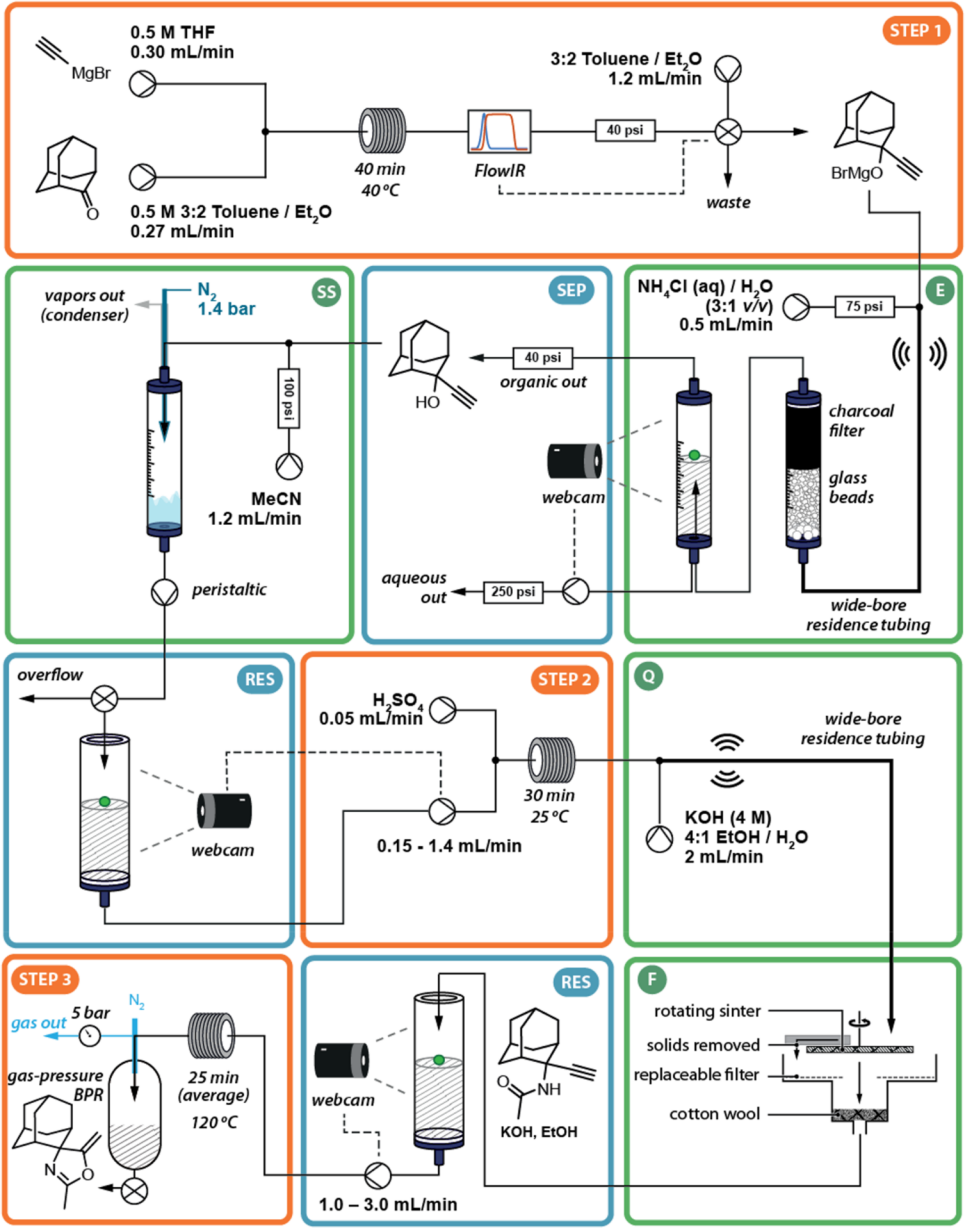
Telescoped eight-step continuous flow synthesis affording a polycyclic intermediate of biological importance. Reproduced from ref (129b). Copyright 2016 American Chemical Society.

Multistep continuous synthesis will require external input to quicken the flow of heterogeneous mixtures through a narrow tubing. An impressive example is shown in Figure 10 detailing the synthesis of a biologically active intermediate from a tricyclic ketone. The complete operational protocol could be executed by a single researcher, who also controlled the machined setup with a low-cost computed-assisted webcam.^129^ The telescoped eight-step route comprises in addition three transformations involving five intermediate downstream processing steps. Sonication was applied to enhance mass transfer in two critical points (steps 1 and 2), thereby increasing overall efficiency.

**Figure 11:**
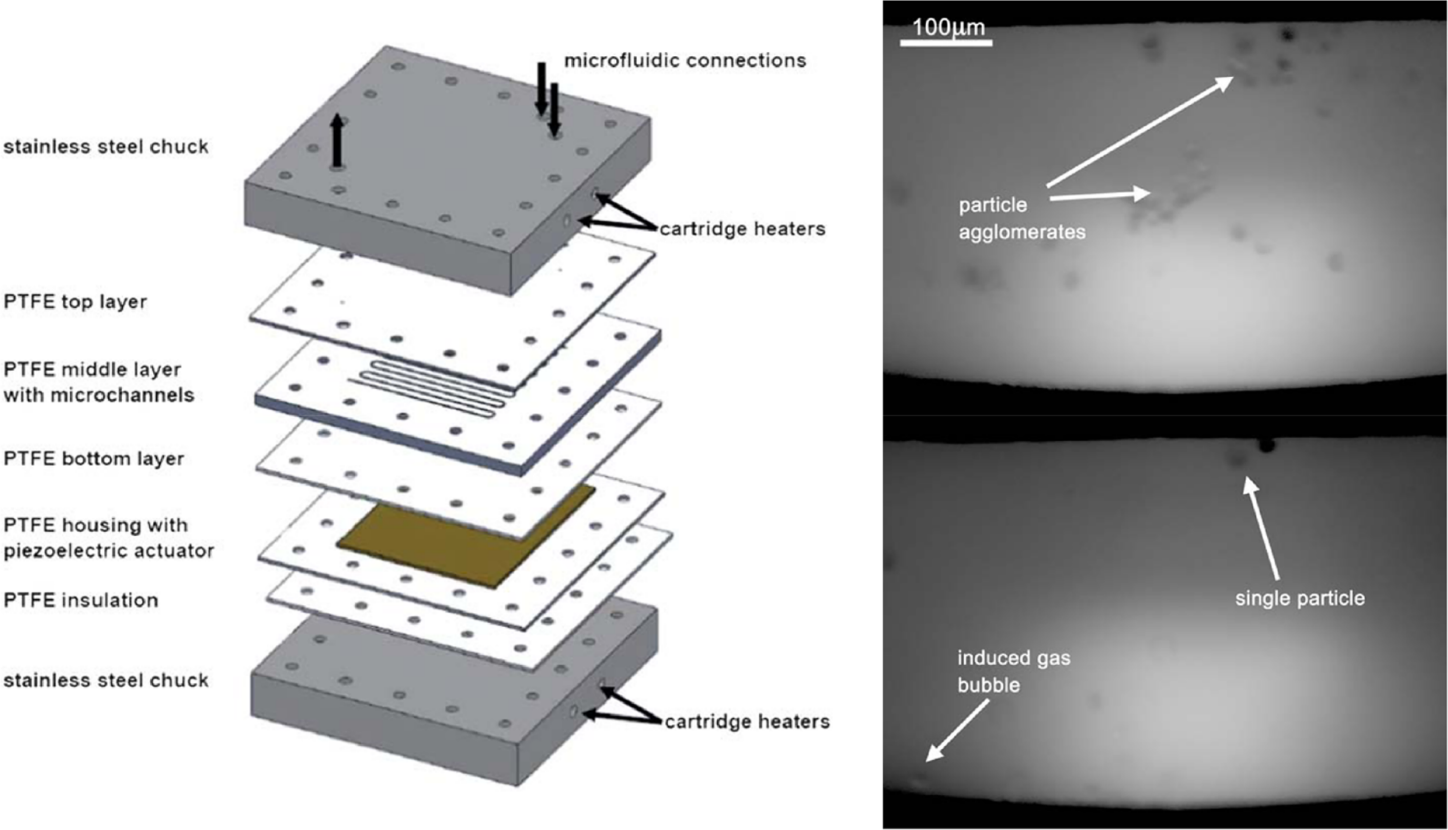
Multilayered Teflon-made microsonoreactor that enables rapid dissolution of solid particles by ultrasonic irradiation as shown by microscopic images. Reproduced with permission from ref (130). Copyright 2011 the Royal Society of Chemistry.

A different design for handling solid-forming reactions involves a multilayered piezoelectric actuator (50 kHz, 300 W) integrated with a Teflon microreactor. The equipment was employed in expeditious and high-yielding (typically >95%) Pd-catalyzed C–N cross-couplings between aryl chlorides and anilines.^130^ Gas bubbles induced by stable cavitation followed by collapse contribute to break and dissolve solid particles (Figure 11).

**Figure 12:**
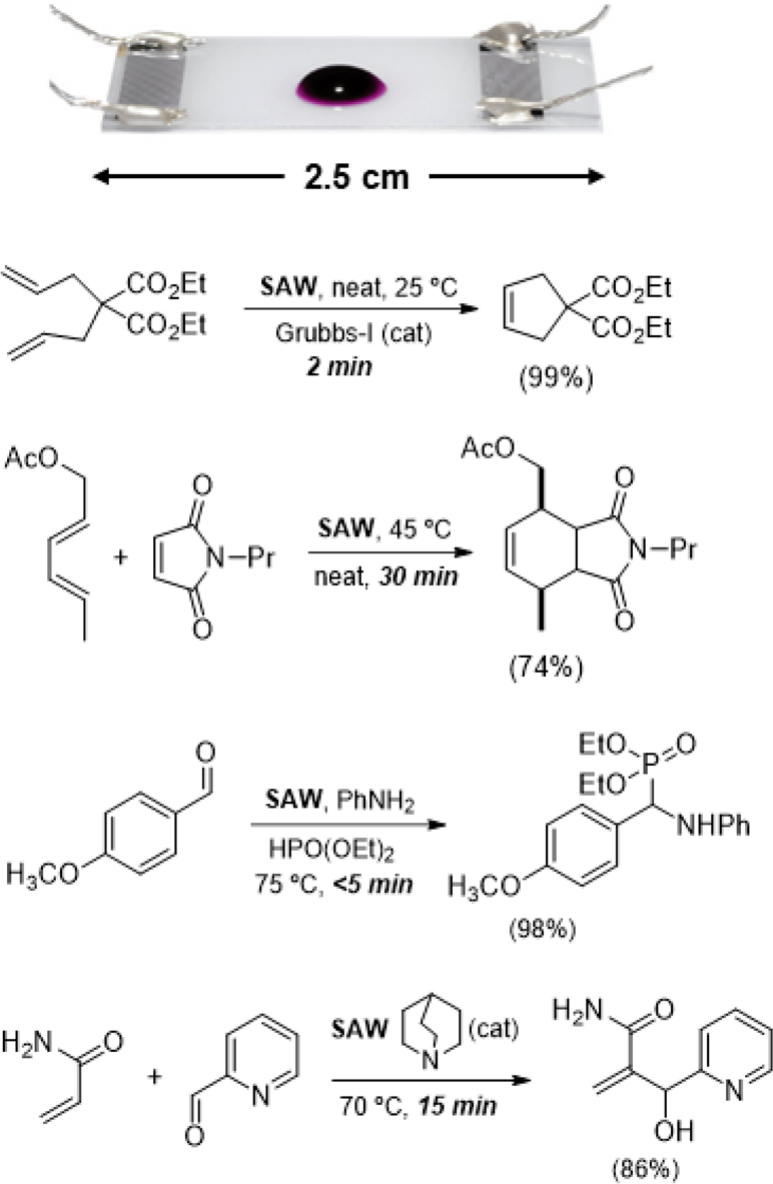
Piezoelectric device employed for some drop reactions (∼40 μL) shown below, irradiated by surface acoustic waves (SAWs) generated by an external oscillating electric field on the piezoelectric material (LiNbO3). Reproduced with permission from ref (131). Copyright 2009 the Royal Society of Chemistry.

Chemical syntheses on a drop scale have been performed using a piezoelectric chip irradiated by surface acoustic waves (SAWs). The miniaturized device consists of a piezoelectric substrate (LiNbO_3_) with two interdigital transducers at each end in the form of 250 nm thick Ti–Al electrodes and then connected to a power source (Figure 12).^131^ By applying an electric field to the piezoelectric material, SAWs are generated as periodic distortions with an amplitude of a few nm and wavelengths of several μm propagating across the surface with the velocity of sound in the solid.^132^ The distance between the electrodes determines the SAW frequency, which may vary from less than 1 MHz to a few GHz. Results collected in Figure 12 were obtained at 20 MHz, usually under net conditions or using a nonvolatile solvent like diethylene glycol, and proceeded at far shorter reaction times than reported conventionally with other energy sources.

Clearly, the working frequencies and acoustic conditions deviate from those of standard power ultrasonics. Cavitation effects should be discarded, as greater intensities would otherwise be required to induce cavitation beyond the MHz threshold. The observed enhancements are largely thermal, and in fact, irradiation with such standing acoustic waves, even for short periods, causes a significant increase in temperature of the drop (tens of degrees). While SAWs have become a promising tool in chemical sensing and nanoparticle manipulation,^133^ from a mechanistic standpoint such soft waves can be an alternative to other droplet generation and aerosol-based methods employing heat or ionization activations. Furthermore, SAWs can inform on catalytic reactions on surfaces. Thus, up to 6-fold rate enhancement has been reported for the catalytic oxidation of CO over platinum films using SAWs under high vacuum.^134^ Although various hypotheses have been suggested, both photoelectron microscopy and dynamic simulations support the thermal activation resulting from energy dissipation along with reversible surface deformation.^135^ The latter can enhance catalytic rates via sorption–desorption process induced by the acoustic oscillations.

## Conclusions and Outlook

*“Oye hijo mío, oye el silencio, Es un silencio onduloso” [“Hear my son, hear the silence, It is a wavy silence”]*

Federico García Lorca (1931)^136^

One could conclude that sonochemistry, dealing with the chemical use of sound in a broad range of frequency and power, has gained a pole position among enabling techniques with an eye on more sustainable protocols. In some ways, however, the discipline cannot be regarded as mature. We should ask questions like, why one or the other product are obtained, whether we can understand how activation happens, and if the use of ultrasound is really justified, rather than claiming another synthetic variation. While ultrasonic activation is rooted in well-established principles of cavitational phenomena, the first action to be taken, which agrees with the rules suggested by Apfel, is *know the equipment you need and how it works*. Reproducible experiments can be run paying attention to a few parameters without worrying about others. In our Perspective, we have given a glimpse on *known facts*. One can anticipate that some reactions and substrates will be sensitive to sonication in either physical or chemical terms. Whether or not ultrasound switches the conventionally assumed mechanism often needs further evidence, where the sonochemical experiment itself can be as informative as other elucidation methods. Attempts to classify sonochemical reactions can result in ill-conceived conclusions if structural and solvent effects that usually accompany cavitation are not investigated in detail. A reductionist approach that merely considers either convergent or divergent results with respect to conventional chemistry may be the key to identifying the tools that are required to understand how the reaction takes place. Whatever the mechanism can be, a different result under sonication is not synonymous with a radical/electron transfer pathway as often and erroneously invoked; two polar mechanisms can compete each other. Perhaps the most divergent results arise from the conceptual framework of *sono-mechanochemistry* as another manifestation of mechanical effects, especially in polymer chains, induced by turbulent solvent flows at the microscale. The issue is complex, although inertial motion in an acoustic field may affect both bonding and energy barriers. As far as we know, cavitation does not involve an electric field component (as once postulated) and our present-day interpretation relies largely upon the *hot spot* theory. Piezoelectric materials, the source of ultrasonic transducers, are susceptible to electric polarization under strain. This peripheral effect can advantageously be exploited in new *mechano-redox processes* that provide salient and unexpected results. A translation of cavitational flow into a continuous operation has enabled further innovation in microfluidics and acoustophoretic devices, which should be part of the future of chemistry. Despite bottlenecks and insufficient knowledge of cavitational effects, the impact of ultrasound-based chemistry is steadily increasing in multiple synthetic and nonsynthetic applications.

## Dedication

This article is dedicated to Jean-Louis Luche (1941–2014) and his family (wife and daughters). Jean-Louis contributed significantly to the current status of synthetic sonochemistry and guided the field with generosity and humility, supporting and inspiring students and friends.

## References

[ref1] Conant J. Tuxedo Park. A Wall Street Tycoon and the Secret Palace of Science that Changed the Course of World War II; Simon & Schuster: New York, 2002; pp 50–51.

[ref2] Luche edited in the late 1990s a multiauthor monograph with a focus on organic sonochemistry and sonosynthesis: Synthetic Organic Sonochemistry; Plenum Press: New York, 1998.

[ref3] Handbook on Applications of Ultrasound. Sonochemistry for Sustainability; Chen D., Sharma S. K., Mudhoo A., Eds.; CRC Press, Inc.: Boca Raton, FL, 2011.

[ref4] *Power Ultrasonics*. Applications of High-Intensity Ultrasound; Gallego-Juárez J. A., Graff K. F., Eds.; Elsevier: Amsterdam, 2015.

[ref5] aChatel G. Sonochemistry. New Opportunities for Green Chemistry; World Scientific Publishing: Singapore, 2017.

[ref6] Lévêque J.-M.; Cravotto G.; Delattre F.; Cintas P. Organic Sonochemistry. Challenges and Perspectives for the 21st-Century; Springer Nature: Cham, Switzerland, 2018.

[ref7] aFor selected reviews and chapters, mostly oriented to organic chemistry/synthesis: Shingare M. S.; Shingate B. B. Ultrasound in synthetic applications and organic chemistry. In Handbook on Applications of Ultrasound. Sonochemistry for Sustainability; Chen D., Sharma S. K., Mudhoo A., Eds.; CRC Press, Inc.: Boca Raton, FL, 2011; pp 213–261.

[ref8] aMason T. J.; Lorimer J. P. Applied Sonochemistry. Uses of Power Ultrasound in Chemistry and Processing; Wiley-VCH: New York, 2002.

[ref9] Cravotto G.; Cintas P. Power ultrasound in organic synthesis: moving cavitational chemistry from academia to innovative and large-scale applications. Chem. Soc. Rev. 2006, 35, 180–196. 10.1039/B503848K. 16444299

[ref10] aLuche J.-L. Sonochemistry. From experiment to theoretical considerations. In Advances in Sonochemistry; Mason T. J., Ed.; JAI Press, Ltd.: London, 1993; Vol. 3, pp 85–124. (b) Luche’s distinction between *true and false sonochemistry*, the former denoting reaction outcomes that arise from cavitation-generated species and the latter stemming largely from enhanced mass transfer in heterogeneous processes, has proven to be useful for classification, although there are numerous borderline situations that hamper generalization.

[ref11] aFor historical perspectives on sonochemistry and cavitation: Bremner D. Historical introduction to sonochemistry. In Advances in Sonochemistry; Mason T. J., Ed.; JAI Press, Ltd.: London, 1990; Vol. 1, pp 1–37.

[ref12] aMcNamara W. B. III; Didenko Y. T.; Suslick K. S. Sonoluminescence temperatures during multi-bubble cavitation. Nature 1999, 401, 772–775. 10.1038/44536.

[ref13] Flannigan D. J.; Suslick K. S. Inertially confined plasma in an imploding bubble. Nat. Phys. 2010, 6, 598–601. 10.1038/nphys1701.

[ref14] For brief remarks on acoustic cavitation, see: Suslick K. S.; Eddingsaas N. C.; Flannigan D. J.; Hopkins S. D.; Xu H. The chemical history of a bubble. Acc. Chem. Res. 2018, 51, 2169–2178. 10.1021/acs.accounts.8b00088. 29771111

[ref15] Schmitt F. O.; Johnson C. H.; Olson A. R. Oxidations promoted by ultrasonic radiation. J. Am. Chem. Soc. 1929, 51, 370–375. 10.1021/ja01377a004.

[ref16] Richards W. T. Supersonic phenomena. Rev. Mod. Phys. 1939, 11, 36–64. 10.1103/RevModPhys.11.36.

[ref17] aXu H.; Zeiger B. W.; Suslick K. S. Sonochemical synthesis of nanomaterials. Chem. Soc. Rev. 2013, 42, 2555–2567. 10.1039/C2CS35282F. 23165883

[ref18] Verhaagen B.; Fernandez Rivas D. Measuring cavitation and its cleaning effect. Ultrason. Sonochem. 2016, 29, 619–628. 10.1016/j.ultsonch.2015.03.009. 25819680

[ref19] aCao H.; Zhang W.; Wang C.; Liang Y. Sonochemical degradation of poly- and perfluoroalkyl substances-A review. Ultrason. Sonochem. 2020, 69, 105245 10.1016/j.ultsonch.2020.105245. 32702636

[ref20] Serna-Galvis E. A.; Montoya-Rodríguez D.; Isaza-Pineda L.; Ibañez M.; Hernández F.; Moncayo-Lasso A.; Torres-Palma R. A. Sonochemical degradation of antibiotics from representative classes. Considerations on structural effects, initial transformation products, antimicrobial activity and matrix. Ultrason. Sonochem. 2019, 50, 157–165. 10.1016/j.ultsonch.2018.09.012. 30241893

[ref21] aSon S.; Kim J. H.; Wang X.; Zhang C.; Yoon S. A.; Shin J.; Sharma A.; Lee M. H.; Cheng L.; Wu J.; Kim J. S. Multifunctional sonosensitizers in sonodynamic cancer therapy. Chem. Soc. Rev. 2020, 49, 3244–3261. 10.1039/C9CS00648F. 32337527

[ref22] Ben-Amots N.; Anbar M. Sonochemistry on primordial earth-its potential role in prebiotic molecular evolution. Ultrason. Sonochem. 2007, 14, 672–675. 10.1016/j.ultsonch.2006.12.007. 17275392

[ref23] aSokol’skaya A.; El’piner I. Chemical synthesis under the action of supersonic waves in a water saturated with gases of a reducing atmosphere. Dokl. Akad. Nauk SSSR 1958, 119, 1180–1182.

[ref24] aAnbar M. Cavitation during impact of liquid water on water. Geochemical implications. Science 1968, 161, 1343–1344. 10.1126/science.161.3848.1343. 17831346

[ref25] Dharmarathne L.; Grieser F. Formation of amino acids on the sonolysis of aqueous solutions containing acetic acid, methane, or carbon dioxide, in the presence of nitrogen gas. J. Phys. Chem. A 2016, 120, 191–199. 10.1021/acs.jpca.5b11858. 26695890

[ref26] Kalson N.-H.; Furman D.; Zeiri Y. Cavitation-induced synthesis of biogenic molecules on primordial earth. ACS Cent. Sci. 2017, 3, 1041–1049. 10.1021/acscentsci.7b00325. 28979946 PMC5620973

[ref27] aKingston C.; Palkowitz M. D.; Takahira Y.; Vantourout J. C.; Peters B. K.; Kawamata Y.; Baran P. S. A survival guide for the “electro-curious”. Acc. Chem. Res. 2020, 53, 72–83. 10.1021/acs.accounts.9b00539. 31823612 PMC6996934

[ref28] Mason T. J.; Cordemans de Meulenaer E. Practical considerations for process optimization. In Synthetic Organic Sonochemistry; Luche J.-L., Ed.; Plenum Press: New York, 1998; Chapter 8, pp 301–329.

[ref29] Crum L. A. Comments on the evolving field of sonochemistry by a cavitation physicist. Ultrason. Sonochem. 1995, 2, S147–S152. 10.1016/1350-4177(95)00018-2.

[ref30] Apfel R. E. Acoustic cavitation. In Methods in Experimental Physics; Edmunds P., Ed.; Academic Press: New York, 1981; Vol. 19, Chapter 7, pp 355–411.

[ref31] Gallego-Juárez J. A.; Graff K. F. Introduction to power ultrasonics. In Power Ultrasonics. Applications of High-Intensity Ultrasound; Gallego-Juárez J. A., Graff K. F., Eds.; Elsevier: Amsterdam, 2015; pp 1–6.

[ref32] Pétrier C.; Jeunet A.; Luche J.-L.; Reverdy G. Unexpected frequency effects on the rate of oxidative processes induced by ultrasound. J. Am. Chem. Soc. 1992, 114, 3148–3152. 10.1021/ja00034a077.

[ref33] aLeighton T. G. The Acoustic Bubble; Academic Press: London, 1994.

[ref34] Wood R. J.; Lee J.; Bussemaker M. J. A parametric review of sonochemistry: control and augmentation of sonochemical activity in aqueous solutions. Ultrason. Sonochem. 2017, 38, 351–370. 10.1016/j.ultsonch.2017.03.030. 28633836

[ref35] Al-Juboori R. A.; Yusaf T.; Bowtell L.; Aravinthan V. Energy characterisation of ultrasonic systems for industrial processes. Ultrasonics 2015, 57, 18–30. 10.1016/j.ultras.2014.10.003. 25455187

[ref36] Mason T. J.; Lorimer J. P.; Bates D. M.; Zhao Y. Dosimetry in sonochemistry: the use of aqueous terephthalate ion as a fluorescence monitor. Ultrason. Sonochem. 1994, 1, 591–595. 10.1016/1350-4177(94)90004-3.

[ref37] Riesz P. Free radical generation by ultrasound in aqueous solutions of volatile and non-volatile solutes. In Advances in Sonochemistry; Mason T. J., Ed.; JAI Press Ltd.: London, 1991; Vol. 2, pp 23–64.

[ref38] Fernandez Rivas D.; Prosperetti A.; Zijlstra A. G.; Lohse D.; Gardeniers H. J. G. E. Efficient sonochemistry through microbubbles generated with micromachined surfaces. Angew. Chem., Int. Ed. 2010, 49, 9699–9701. 10.1002/anie.201005533. 21064078

[ref39] Lee J.; Yasui K.; Ashokkumar M.; Kentish S. E. Quantification of cavitation activity by sonoluminescence to study the sonocrystallization process under different ultrasound parameters. Cryst. Growth Des. 2018, 18, 5108–5115. 10.1021/acs.cgd.8b00547.

[ref40] In a sonochemical context, the terms *convergent* and *divergent* suggested by Luche too, are intended to mean “the same” and “not the same”, respectively, when silent and irradiated processes are compared each other. In organic synthesis, the usual connotation given to a *convergent approach* involves coupling of fragments with similar complexity and/or functionality, thereby assembling the target molecule in a convergent plan (as opposed to a stepwise linear strategy); for a recent overview, see: Gao Y.; Ma D. In Pursuit of Synthetic Efficiency: Convergent Approaches. Acc. Chem. Res. 2021, 54, 569–582. 10.1021/acs.accounts.0c00727. 33448789

[ref41] Sinisterra J. V.; Fuentes A.; Marinas J. M. Barium hydroxide as catalyst in organic reactions. 17. Interfacial solid-liquid Wittig-Horner reaction under sonochemical conditions. J. Org. Chem. 1987, 52, 3875–3879. 10.1021/jo00226a029.

[ref42] Makino K.; Mossoba M. M.; Riesz P. Chemical effects of ultrasound on aqueous solutions. Evidence for hydroxyl and hydrogen free radicals (.cntdot.OH and. cntdot.H) by spin trapping. J. Am. Chem. Soc. 1982, 104, 3537–3539. 10.1021/ja00376a064.

[ref43] Einhorn C.; Luche J.-L. Ready preparation of sugar acetals under ultrasonic irradiation. Carbohydr. Res. 1986, 155, 258–261. 10.1016/S0008-6215(00)90155-1.

[ref44] Low C. M. R. Ultrasound in synthesis: natural products and supersonic reactions?. Ultrason. Sonochem. 1995, 2, S153–S163. 10.1016/1350-4177(95)00017-Z.

[ref45] Collum D. B.; McNeil A. J.; Ramirez A. Lithium diisopropylamide: solution kinetics and implications for organic synthesis. Angew. Chem., Int. Ed. 2007, 46, 3002–3017. 10.1002/anie.200603038. 17387670

[ref46] Szostak M.; Spain M.; Procter D. J. Preparation of samarium(II) iodide: quantitative evaluation of the effect of water, oxygen, and peroxide content, preparative methods, and the activation of samarium metal. J. Org. Chem. 2012, 77, 3049–3059. 10.1021/jo300135v. 22375820

[ref47] De Nicola A.; Einhorn J.; Luche J.-L. An easy preparation of hindered lithium amides. J. Chem. Res., Synop. 1991, 278.

[ref48] Ley S. V.; Low C. M. R. Ultrasound in Synthesis; Springer: Heidelberg, 1989; pp 105–118.

[ref49] For an analysis and discussion of some representative examples, see: Luche J.-L.; Cintas P. Can sonication modify the regio- and stereoselectivities of organic reactions? In Advances in Sonochemistry; Mason T. J., Ed.; JAI Press, Ltd.: London, 1999; Vol. 5, pp 147–174.

[ref50] Jayarajan R.; Chandrashekar H. B.; Dalvi A. K.; Maiti D. Ultrasound-facilitated direct *meta*-C-H functionalization of arenes: a time-economical strategy under ambient temperature with improved yield and selectivity. Chem. - Eur. J. 2020, 26, 11426–11430. 10.1002/chem.202001757. 32289187

[ref51] Cintas P.; Palmisano G.; Cravotto G. Power ultrasound in metal-assisted synthesis: from classical Barbier-like reactions to click chemistry. Ultrason. Sonochem. 2011, 18, 836–841. 10.1016/j.ultsonch.2010.11.020. 21216171

[ref52] Cintas P.; Barge A.; Tagliapietra S.; Boffa L.; Cravotto G. Alkyne-azide click reaction catalyzed by metallic copper under ultrasound. Nat. Protoc. 2010, 5, 607–611. 10.1038/nprot.2010.1. 20203675

[ref53] May P. A.; Moore J. S. Polymer mechanochemistry: techniques to generate molecular force via elongational flows. Chem. Soc. Rev. 2013, 42, 7497–7506. 10.1039/c2cs35463b. 23306817

[ref54] Cravotto G.; Gaudino E. C.; Cintas P. On the mechanochemical activation by ultrasound. Chem. Soc. Rev. 2013, 42, 7521–7534. 10.1039/c2cs35456j. 23321794

[ref55] Li J.; Nagamani C.; Moore J. S. Polymer mechanochemistry: from destructive to productive. Acc. Chem. Res. 2015, 48, 2181–2190. 10.1021/acs.accounts.5b00184. 26176627

[ref56] Cintas P.; Cravotto G.; Barge A.; Martina K. Interplay between mechanochemistry and sonochemistry. In Polymer Mechanochemistry-Topics in Current Chemistry, Vol. 369; Boulatov R., Ed.; Springer: Heidelberg, 2015; pp 239–284. 10.1007/128_2014_623 25860254

[ref57] Mason T. J.; Cobley A. J.; Graves J. E.; Morgan D. New evidence for the inverse dependence of mechanical and chemical effects on the frequency of ultrasound. Ultrason. Sonochem. 2011, 18, 226–230. 10.1016/j.ultsonch.2010.05.008. 20605105

[ref58] Nguyen T. T.; Asakura Y.; Koda S.; Yasuda K. Dependence of cavitation, chemical effect and mechanical effect thresholds on ultrasonic frequency. Ultrason. Sonochem. 2017, 39, 301–306. 10.1016/j.ultsonch.2017.04.037. 28732949

[ref59] Boldyrev V. V. Mechanochemistry and sonochemistry. Ultrason. Sonochem. 1995, 2, S143–S145. 10.1016/1350-4177(95)00019-3.

[ref60] Nguyen T. Q.; Liang O. Z.; Kausch H. H. Kinetics of ultrasonic and transient elongational flow degradation: a comparative study. Polymer 1997, 38, 3783–3793. 10.1016/S0032-3861(96)00950-0.

[ref61] For a good overview of theoretical interpretations of polymer mechanochemistry in sonicated solutions, see: Akbulatov S.; Boulatov R. Experimental polymer mechanochemistry and its interpretational frameworks. ChemPhysChem 2017, 18, 1422–1450. 10.1002/cphc.201601354. 28256793

[ref62] Hickenboth C. R.; Moore J. S.; White S. R.; Sottos N. R.; Baudry J.; Wilson S. R. Biasing reaction pathways with mechanical force. Nature 2007, 446, 423–427. 10.1038/nature05681. 17377579

[ref63] Ribas-Arino J.; Marx D. Covalent mechanochemistry: theoretical concepts and computational tools with applications to molecular nanomechanics. Chem. Rev. 2012, 112, 5412–5487. 10.1021/cr200399q. 22909336

[ref64] Stauch T.; Dreuw A. Advances in quantum mechanochemistry: electronic structure methods and force analysis. Chem. Rev. 2016, 116, 14137–14180. 10.1021/acs.chemrev.6b00458. 27767298

[ref65] aGarcia-Manyes S.; Beedle A. E. M. Steering chemical reactions with force. Nat. Rev. Chem. 2017, 1, 0083 10.1038/s41570-017-0083.

[ref66] Andersen J. M.; Mack J. Decoupling the Arrhenius equation via mechanochemistry. Chem. Sci. 2017, 8, 5447–5453. 10.1039/C7SC00538E. 28970924 PMC5609516

[ref67] aGogate P. R.; Prajapat A. L. Depolymerization using sonochemical reactors: a critical review. Ultrason. Sonochem. 2015, 27, 480–494. 10.1016/j.ultsonch.2015.06.019. 26186870

[ref68] aBerkowski K. L.; Potisek S. L.; Hickenboth C. R.; Moore J. S. Ultrasound-induced site-specific cleavage of azo-functionalized poly(ethylene glycol). Macromolecules 2005, 38, 8975–8978. 10.1021/ma051394n.

[ref69] See, for instance: Nakamura E.; Imanishi Y.; Machii D. Sonochemical initiation of radical chain reactions. Hydrostannation and hydroxystannation of C-C multiple bonds. J. Org. Chem. 1994, 59, 8178–8186. 10.1021/jo00105a039.

[ref70] Bowser B. H.; Craig S. L. Empowering mechanochemistry with multi-mechanophore polymer architectures. Polym. Chem. 2018, 9, 3583–3593. 10.1039/C8PY00720A.

[ref71] Chen Z.; Mercer J. A. M.; Zhu X.; Romaniuk J. A. H.; Pfattner R.; Cegelski L.; Martinez T. J.; Burns N. Z.; Xia Y. Mechanochemical unzipping of insulating polyladderene to semiconducting polyacetylene. Science 2017, 357, 475–479. 10.1126/science.aan2797. 28774923

[ref72] aChen Z.; Zhu X.; Yang J.; Mercer J. A. M.; Burns N. Z.; Martinez T. J.; Xia Y. The cascade unzipping of ladderane reveals dynamic effects in mechanochemistry. Nat. Chem. 2020, 12, 302–309. 10.1038/s41557-019-0396-5. 31907403

[ref73] Huang W.; Wu X.; Gao X.; Yu Y.; Lei H.; Zhu Z.; Shi Y.; Chen Y.; Qin M.; Wang W.; Cao Y. Maleimide-thiol adducts stabilized through stretching. Nat. Chem. 2019, 11, 310–319. 10.1038/s41557-018-0209-2. 30718898

[ref74] Shi Z.; Song Q.; Göstl R.; Herrmann A. Mechanochemical activation of disulfide-based multifunctional polymers for theranostic drug release. Chem. Sci. 2021, 12, 1668–1674. 10.1039/D0SC06054B. PMC8179261 34163927

[ref75] aHuo S.; Zhao P.; Shi Z.; Zou M.; Yang X.; Warszawik E.; Loznik M.; Göstl R.; Herrmann A. Mechanochemical bond scission for the activation of drugs. Nat. Chem. 2021, 13, 131–139. 10.1038/s41557-020-00624-8. 33514936

[ref76] Lattwein K. R.; Shekhar H.; Kouijzer J. J. P.; Van Wamel W. J. B.; Holland C. K.; Kooiman K. Sonobactericide: an emerging treatment strategy for bacterial infections. Ultrasound Med. Biol. 2020, 46, 193–215. 10.1016/j.ultrasmedbio.2019.09.011. 31699550 PMC9278652

[ref77] Chettab K.; Mestas J.-L.; Lafond M.; Saadna D. E.; Lafon C.; Dumontet C. Doxorubicin delivery into tumor cells by stable cavitation without contrast agents. Mol. Pharmaceutics 2017, 14, 441–447. 10.1021/acs.molpharmaceut.6b00880. 28107023

[ref78] aCádiz Bedini A. P.; Muthmann S.; Allgaier J.; Bittkau K.; Finger F.; Carius R. Liquid hydridosilane precursor prepared from cyclopentasilane via sonication at low temperature without the action of light. Ultrason. Sonochem. 2017, 34, 289–293. 10.1016/j.ultsonch.2016.05.039. 27773248

[ref79] aCravotto G.; Borretto E.; Oliverio M.; Procopio A.; Penoni A. Organic reactions in water or biphasic aqueous systems under sonochemical conditions. A review on catalytic effects. Catal. Commun. 2015, 63, 2–9. 10.1016/j.catcom.2014.12.014.

[ref80] Jiménez-González C.; Constable D. J. C.; Ponder C. S. Evaluating the greenness of chemical processes and products in the pharmaceutical industry-a green metrics primer. Chem. Soc. Rev. 2012, 41, 1485–1498. 10.1039/C1CS15215G. 22076593

[ref81] aFegade S. L.; Trembly J. P. Misinterpretation of Green chemistry. Ultrason. Sonochem. 2017, 37, 686–687. 10.1016/j.ultsonch.2015.04.007. 25952830

[ref82] Li C. J.; Chen L. Organic chemistry in water. Chem. Soc. Rev. 2006, 35, 68–82. 10.1039/B507207G. 16365643

[ref83] Ando T.; Kimura T. Ultrasonic organic synthesis involving non-metal solids. In Advances in Sonochemistry; Mason T. J., Ed.; JAI Press, Inc.: London, 1991; Vol. 2, pp 211–251 and references cited therein.

[ref84] aTuulmets A.; Hagu H.; Salmar S.; Cravotto G.; Järv J. Ultrasonic evidence of hydrophobic interactions. Effect of ultrasound on benzoin condensation and some other reactions in aqueous ethanol. J. Phys. Chem. B 2007, 111, 3133–3138. 10.1021/jp0682199. 17388456

[ref85] aGulajski L.; Sledz P.; Lupa A.; Grela K. Olefin metathesis in water using acoustic emulsification. Green Chem. 2008, 10, 271–274. 10.1039/b719493e.

[ref86] Jaiswal P. K.; Sharma V.; Prikhodko J.; Mashevskaya I. V.; Chaudhary S. On water ultrasound-assisted one-pot efficient synthesis of functionalized 2-oxo-benzo[1,4]oxazines: first application to the synthesis of anticancer indole alkaloid Cephalandole A. Tetrahedron Lett. 2017, 58, 2077–2083. 10.1016/j.tetlet.2017.03.048.

[ref87] Rogozinska M.; Adamkiewicz A.; Mlynarski J. Efficient “on water” organocatalytic protocol for the synthesis of optically pure warfarin anticoagulant. Green Chem. 2011, 13, 1155–1157. 10.1039/c1gc15118e.

[ref88] Lee J.; Kentish S.; Matula T. J.; Ashokkumar M. Effect of surfactants on inertial cavitation activity in pulsed acoustic field. J. Phys. Chem. B 2005, 109, 16860–16865. 10.1021/jp0533271. 16853145

[ref89] More P. A.; Shankarling G. S. Energy efficient Pfitzinger reaction: a novel strategy using a surfactant catalyst. New J. Chem. 2017, 41, 12380–12383. 10.1039/C7NJ01937H.

[ref90] Kamble S.; Kumbhar A.; Rashinkar G.; Barge M.; Salunkhe R. Ultrasound promoted efficient and green synthesis of β-amino carbonyl compounds in aqueous hydrotropic medium. Ultrason. Sonochem. 2012, 19, 812–815. 10.1016/j.ultsonch.2011.12.001. 22230101

[ref91] Saleh T. S.; Al-Bogami A. S.; Mekky A. E. M.; Alkhathlan H. Z. Sonochemical synthesis of novel pyrano[3,4-*e*][1,3]oxazines: a green protocol. Ultrason. Sonochem. 2017, 36, 474–480. 10.1016/j.ultsonch.2016.12.015. 28069235

[ref92] Fillion H.; Luche J.-L. Cycloadditions. In Synthetic Organic Sonochemistry; Luche J.-L., Ed.; Plenum Press: New York, 1998; pp 91–106 and references cited therein.

[ref93] aBauld N. L. Cation radical cycloadditions and related sigmatropic reactions. Tetrahedron 1989, 45, 5307–5363. 10.1016/S0040-4020(01)89486-2.

[ref94] aCabello N.; Cintas P.; Luche J.-L. Sonochemical effects in the addition of furan to masked *ortho*-benzoquinones. Ultrason. Sonochem. 2003, 10, 25–31. 10.1016/S1350-4177(02)00103-7. 12457947

[ref95] For a recent critical review, see: Schmidt F.; Cokoja M. Supramolecular concepts for the biphasic epoxidation of olefins using aqueous hydrogen peroxide. Green Chem. 2021, 23, 708–722. 10.1039/D0GC03580G.

[ref96] Chatel G.; Goux-Henry C.; Mirabaud A.; Rossi T.; Kardos N.; Andrioletti B.; Draye M. H_2_O_2_/NaHCO_3_-Mediated enantioselective epoxidation of olefins in NTf_2_-based ionic liquids and under ultrasound. J. Catal. 2012, 291, 127–132. 10.1016/j.jcat.2012.04.016.

[ref97] Cousin T.; Chatel G.; Kardos N.; Andrioletti B.; Draye M. High frequency ultrasound as a tool for elucidating mechanistic elements of cis-cyclooctene epoxidation with aqueous hydrogen peroxide. Ultrason. Sonochem. 2019, 53, 120–125. 10.1016/j.ultsonch.2018.12.038. 30686597

[ref98] Calcio Gaudino E.; Cravotto G.; Manzoli M.; Tabasso S. Sono- and mechanochemical technologies in the catalytic conversion of biomass. Chem. Soc. Rev. 2021, 50, 1785–1812. 10.1039/D0CS01152E. 33313620

[ref99] Amaniampong P. N.; Trinh Q. T.; De Oliveira Vigier K.; Dao D. Q.; Tran N. H.; Wang Y.; Sherburne M. P.; Jérôme F. Synergistic effect of high-frequency ultrasound with cupric oxide catalyst resulting in a selectivity switch in glucose oxidation under argon. J. Am. Chem. Soc. 2019, 141, 14772–14779. 10.1021/jacs.9b06824. 31450888

[ref100] Newnham R. E. Properties of Materials. Anisotropy, Symmetry, Structure; Oxford University Press: Oxford, UK, 2005; pp 87–102.

[ref101] Pardo L. Piezoelectric ceramic materials for power ultrasonic transducers. In Power Ultrasonics. Applications of High-Intensity Ultrasound; Gallego-Juárez J. A., Graff K. F., Eds.; Elsevier: Amsterdam, 2015; pp 101–125.

[ref102] Li F.; Cabral M. J.; Xu B.; Cheng Z.; Dickey E. C.; LeBeau J. M.; Wang J.; Luo J.; Taylor S.; Hackenberger W.; Bellaiche L.; Xu Z.; Chen L.-Q.; Shrout T. R.; Zhang S. Giant piezoelectricity of Sm-doped Pb(Mg_1/3_Nb_2/3_)O_3_-PbTiO_3_ single crystals. Science 2019, 364, 264–268. 10.1126/science.aaw2781. 31000659

[ref103] Graff K. F. Power ultrasonic transducers: principles and design. In Power Ultrasonics. Applications of High-Intensity Ultrasound; Gallego-Juárez J. A., Graff K. F., Eds.; Elsevier: Amsterdam, 2015; pp 127–158.

[ref104] Hong K.-S.; Xu H.; Konishi H.; Li X. Direct water splitting through vibrating piezoelectric microfibers in water. J. Phys. Chem. Lett. 2010, 1, 997–1002. 10.1021/jz100027t.

[ref105] Morosini V.; Chave T.; Virot M.; Moisy P.; Nikitenko S. I. Sonochemical water splitting in the presence of powdered metal oxides. Ultrason. Sonochem. 2016, 29, 512–516. 10.1016/j.ultsonch.2015.11.006. 26558997

[ref106] Vedadi M. H.; Haas S. Mechano-chemical pathways to H_2_O and CO_2_ spliting. Appl. Phys. Lett. 2011, 99, 154105 10.1063/1.3650695.

[ref107] aWang Z.; Pan X.; Yan J.; Dadashi-Silab S.; Xie G.; Zhang J.; Wang Z.; Xia H.; Matyjaszewski K. Temporal control in mechanically controlled atom transfer radical polymerization using low ppm of Cu catalyst. ACS Macro Lett. 2017, 6, 546–549. 10.1021/acsmacrolett.7b00152. 35610875

[ref108] Mohapatra H.; Kleiman M.; Esser-Kahn A. P. Mechanically controlled radical polymerization initiated by ultrasound. Nat. Chem. 2017, 9, 135–139. 10.1038/nchem.2633.

[ref109] Mohapatra H.; Ayarza J.; Sanders E. C.; Scheuermann A. M.; Griffin P. J.; Esser-Kahn A. P. Ultrasound promoted step-growth polymerization and polymer crosslinking via copper catalyzed azide-alkyne “click” reaction. Angew. Chem., Int. Ed. 2018, 57, 11208–11212. 10.1002/anie.201804451. 29992680

[ref110] Kubota K.; Pang Y.; Miura A.; Ito H. Redox reactions of small organic molecules using ball milling and piezoelectric materials. Science 2019, 366, 1500–1504. 10.1126/science.aay8224. 31857482

[ref111] Pang Y.; Lee J. W.; Kubota K.; Ito H. Solid-state radical C-H trifluoromethylation reactions using ball milling and piezoelectric materials. Angew. Chem., Int. Ed. 2020, 59, 22570–22576. 10.1002/anie.202009844. 32914933

[ref112] Huang G.; Chen S.; Dai C.; Sun L.; Sun W.; Tang Y.; Xiong F.; He R.; Ma H. Effects of ultrasound on microbial growth and enzyme activity. Ultrason. Sonochem. 2017, 37, 144–149. 10.1016/j.ultsonch.2016.12.018. 28427617

[ref113] Yoon J.; Kim J.; Tieves F.; Zhang W.; Alcalde M.; Hollmann F.; Park C. B. Piezobiocatalysis: ultrasound-driven enzymatic oxyfunctionalization of C-H bonds. ACS Catal. 2020, 10, 5236–5242. 10.1021/acscatal.0c00188.

[ref114] Takenaka T. Bismuth-based piezoelectric ceramics. In Piezoelectric and Acoustic Materials for Transducer Applications; Safari A., Akdogan E. K., Eds.; Springer: Boston, 2008; pp 103–130.

[ref115] For a previous study merging the action of piezoelectric BiOCl and ultrasound, see: Shao D.; Zhang L.; Sun S.; Wang W. Oxygen reduction reaction for generating H_2_O_2_ through a piezo-catalytic process over bismuth oxychloride. ChemSusChem 2018, 11, 527–531. 10.1002/cssc.201702405. 29316272

[ref116] For another piezoelectric titanate activated by ultrasound to produce hydroxyl radicals, see: Ling J.; Wang K.; Wang Z.; Huang H.; Zhang G. Enhanced piezoelectric-induced catalysis of SrTiO_3_ nanocrystal with well-defined facets under ultrasonic vibration. Ultrason. Sonochem. 2020, 61, 104819 10.1016/j.ultsonch.2019.104819. 31669844

[ref117] aLi J.; Ballmer S. G.; Gillis E. P.; Fujii S.; Schmidt M. J.; Palazzolo A. M. E.; Lehmann J. W.; Morehouse G. F.; Burke M. D. Synthesis of many different types of organic small molecules using an automated process. Science 2015, 347, 1221–1226. 10.1126/science.aaa5414. 25766227 PMC4687482

[ref118] aKlucznik T.; Mikulak-Klucznik B.; McCormack M. P.; Lima H.; Szymku S.; Bhowmick M.; Molga K.; Zhou Y.; Rickershauser L.; Gajewska E. P.; Toutchkine A.; Dittwald P.; Startek M. P.; Kirkovits G. J.; Roszak R.; Adamski A.; Sieredzinska B.; Mrksich M.; Trice S. L. J.; Grzybowski B. A. Efficient synthesis of diverse medicinally relevant targets planned by computer and executed in the laboratory. Chem. 2018, 4, 1–11. 10.1016/j.chempr.2018.02.002.

[ref119] aGogate P. R.; Sutkar V. S.; Pandit A. B. Sonochemical reactors: important design and scale up considerations with a special emphasis on heterogeneous systems. Chem. Eng. J. 2011, 166, 1066–1082. 10.1016/j.cej.2010.11.069.

[ref120] aCintas P.; Mantegna S.; Gaudino E. C.; Cravotto G. A new pilot flow reactor for high-intensity ultrasound irradiation. Application to the synthesis of biodiesel. Ultrason. Sonochem. 2010, 17, 985–989. 10.1016/j.ultsonch.2009.12.003. 20060353

[ref121] aFernandez Rivas D.; Cintas P.; Gardeniers H. J. G. E. Merging microfluidics and sonochemistry: towards greener and more efficient micro-sono-reactors. Chem. Commun. 2012, 48, 10935–10947. 10.1039/c2cc33920j. 23001310

[ref122] Li Y.; Liu X.; Huang Q.; Ohta A. T.; Arai T. Bubbles in microfluidics: an all-purpose tool for micromanipulation. Lab Chip 2021, 21, 1016–1035. 10.1039/D0LC01173H. 33538756

[ref123] Campos K. R.; Coleman P. J.; Alvarez J. C.; Dreher S. D.; Garbaccio R. M.; Terrett N. K.; Tillyer R. D.; Truppo M. D.; Parmee E. R. The importance of synthetic chemistry in the pharmaceutical industry. Science 2019, 363, eaat0805 10.1126/science.aat0805. 30655413

[ref124] Gutmann B.; Cantillo D.; Kappe C. O. Continuous flow technology-a tool for the safe manufacturing of active pharmaceutical ingredients. Angew. Chem., Int. Ed. 2015, 54, 6688–6728. 10.1002/anie.201409318. 25989203

[ref125] Cantillo D.; Damm M.; Dallinger D.; Bauser M.; Berger M.; Kappe C. O. Sequential nitration/hydrogenation protocol for the synthesis of triaminophloroglucinol: safe generation and use of an explosive intermediate under continuous-flow conditions. Org. Process Res. Dev. 2014, 18, 1360–1366. 10.1021/op5001435.

[ref126] aJensen K. F.; Reizman B. J.; Newman S. G. Tools for chemical synthesis in microsystems. Lab Chip 2014, 14, 3206–3212. 10.1039/C4LC00330F. 24865228

[ref127] Aljbour S.; Yamada H.; Tagawa T. Ultrasound-assisted phase-transfer catalysis in a capillary microreactor. Chem. Eng. Process. 2009, 48, 1167–1172. 10.1016/j.cep.2009.04.004.

[ref128] Banaszak-Léonard E.; Mangin F.; Len C. Barton decarboxylation under ultrasonic continuous flow. New J. Chem. 2016, 40, 7414–7420. 10.1039/C6NJ01368F.

[ref129] aIngham R. J.; Battilochio C.; Fitzpatrick D. E.; Sliwinski E.; Hawkins J. M.; Ley S. V. A systems approach towards intelligent and self-controlling platform for integrated continuous reaction sequences. Angew. Chem., Int. Ed. 2015, 54, 144–148. 10.1002/anie.201409356. PMC4502965 25377747

[ref130] Kuhn S.; Noël T.; Gu L.; Heider P. L.; Jensen K. F. A Teflon microreactor with integrated piezoelectric actuator to handle solid forming reactions. Lab Chip 2011, 11, 2488–2492. 10.1039/c1lc20337a. 21701722

[ref131] Kulkarni K.; Friend J.; Yeo L.; Perlmutter P. Surface acoustic waves as an emerging source for drop scale synthetic chemistry. Lab Chip 2009, 9, 754–755. 10.1039/b819217k. 19255655

[ref132] aKuznetsova I. E.; Zaitsev B. D.; Joshi S. G.; Borodina I. A. Investigation of acoustic waves in thin plates of lithium niobate and lithium tantalate. IEEE Trans. Ultrasonics Ferroelectrics & Frequency 2001, 48, 322–328. 10.1109/58.896145. 11367801

[ref133] aLin S.-C. S.; Mao X.; Huang T. J. Surface acoustic wave (SAW) acoustophoresis: now and beyond. Lab Chip 2012, 12, 2766–2770. 10.1039/c2lc90076a. 22781941 PMC3992433

[ref134] aKelling S.; Cerasari S.; Rotermund H. H.; Ertl G.; King D. A. A photoemission electron microscopy (PEEM) study of the effect of surface acoustic waves on catalytic CO oxidation over Pt{110}. Chem. Phys. Lett. 1998, 293, 325–330. 10.1016/S0009-2614(98)00811-2.

[ref135] aWu C.; Zaitsev V. Y.; Zhigilei L. V. Acoustic enhancement of surface diffusion. J. Phys. Chem. C 2013, 117, 9252–9258. 10.1021/jp400884d.

[ref136] García Lorca F. Romancero Gitano-Poema del Cante Jondo; Anaya: Madrid, 2006.

